# Mapping eGFR loci to the renal transcriptome and phenome in the VA Million Veteran Program

**DOI:** 10.1038/s41467-019-11704-w

**Published:** 2019-08-26

**Authors:** Jacklyn N. Hellwege, Digna R. Velez Edwards, Ayush Giri, Chengxiang Qiu, Jihwan Park, Eric S. Torstenson, Jacob M. Keaton, O. D. Wilson, Cassianne Robinson-Cohen, Cecilia P. Chung, Christianne L. Roumie, Derek Klarin, Scott M. Damrauer, Scott L. DuVall, Edward Siew, Elvis A. Akwo, Matthias Wuttke, Mathias Gorski, Man Li, Yong Li, J. Michael Gaziano, Peter W. F. Wilson, Philip S. Tsao, Christopher J. O’Donnell, Csaba P. Kovesdy, Cristian Pattaro, Anna Köttgen, Katalin Susztak, Todd L. Edwards, Adriana M. Hung

**Affiliations:** 1 Biomedical Laboratory Research and Development, Tennessee Valley Healthcare System (626)/Vanderbilt University, Nashville, TN USA; 2 Division of Genetic Medicine, Department of Medicine, Vanderbilt Genetics Institute, Vanderbilt University Medical Center, Nashville, TN USA; 3 Department of Obstetrics & Gynecology, Vanderbilt Genetics Institute, Vanderbilt Epidemiology Center, Vanderbilt University Medical Center, Nashville, TN USA; 4 Department of Biomedical Informatics, Vanderbilt University Medical Center, Nashville, TN USA; 5 Department of Medicine, Renal Electrolyte and Hypertension Division, University of Pennsylvania, Philadelphia, PA USA; 6 Division of Epidemiology, Department of Medicine, Vanderbilt Genetics Institute, Vanderbilt University Medical Center, Nashville, TN USA; 7 Division of Nephrology and Hypertension, Department of Medicine, Vanderbilt University Medical Center, Nashville, TN USA; 8 Divisions of Rheumatology and Clinical Pharmacology, Department of Medicine, Vanderbilt University Medical Center, Nashville, TN USA; 9 Veteran Affairs Administration Tennessee Valley VA Health Care System Geriatric Research Education Clinical Center (GRECC), Nashville, TN USA; 10 Department of Medicine, Vanderbilt University Medical Center, Nashville, TN USA; 11 VA Boston Health Care System, Boston, MA USA; 12 Center for Genomic Medicine, Massachusetts General Hospital, Harvard Medical School, Boston, MA USA; 13 Program in Medical and Population Genetics, Broad Institute of Harvard and MIT, Cambridge, MA USA; 14 Department of Surgery, Massachusetts General Hospital, Harvard Medical School, Boston, MA USA; 15 Department of Surgery, Corporal Michael Crescenz VA Medical Center, Philadelphia, PA USA; 16 Department of Surgery, Perelman School of Medicine, University of Pennsylvania, Philadelphia, PA USA; 17 VA Salt Lake City Health Care System, Salt Lake City, UT USA; 18 University of Utah School of Medicine, Salt Lake City, UT USA; 19 Institute of Genetic Epidemiology, Department of Biometry, Epidemiology and Medical Bioinformatics, Faculty of Medicine and Medical Centre—University of Freiburg, Freiburg, Germany; 20 Department of Genetic Epidemiology, Institute of Epidemiology and Preventive Medicine, University of Regensburg, Regensburg, Germany; 21 Department of Nephrology, University Hospital Regensburg, Regensburg, Germany; 22 Division of Nephrology, Department of Internal Medicine, University of Utah School of Medicine, Salt Lake City, UT USA; 23 Massachusetts Veterans Epidemiology Research and Information Center (MAVERIC), VA Boston Healthcare System, Boston, MA USA; 24 Atlanta VA Medical Center, Atlanta, GA USA; 25 Emory Clinical Cardiovascular Research Institute, Atlanta, GA USA; 26 VA Palo Alto Health Care System, Palo Alto, CA USA; 27 Department of Medicine, Stanford University School of Medicine, Stanford, CA USA; 28 Section of Cardiology and Department of Medicine, Brigham and Women’s Hospital, Harvard Medical School, Boston, MA USA; 29 Nephrology Section, Memphis VA Medical Center, Memphis, TN USA; 30 Division of Nephrology, University of Tennessee Health Science Center, Memphis, TN USA; 31 Institute for Biomedicine, Eurac Research, Bolzano, Italy; 32 Department of Epidemiology, Johns Hopkins Bloomberg School of Public Health, Baltimore, MD USA

**Keywords:** Chronic kidney disease, Kidney, Gene expression

## Abstract

Chronic kidney disease (CKD), defined by low estimated glomerular filtration rate (eGFR), contributes to global morbidity and mortality. Here we conduct a transethnic Genome-Wide Association Study of eGFR in 280,722 participants of the Million Veteran Program (MVP), with replication in 765,289 participants from the Chronic Kidney Disease Genetics (CKDGen) Consortium. We identify 82 previously unreported variants, confirm 54 loci, and report interesting findings including association of the sickle cell allele of betaglobin among non-Hispanic blacks. Our transcriptome-wide association study of kidney function in healthy kidney tissue identifies 36 previously unreported and nine known genes, and maps gene expression to renal cell types. In a Phenome-Wide Association Study in 192,868 MVP participants using a weighted genetic score we detect associations with CKD stages and complications and kidney stones. This investigation reinterprets the genetic architecture of kidney function to identify the gene, tissue, and anatomical context of renal homeostasis and the clinical consequences of dysregulation.

## Abstract (web summary)

Persistently low levels of estimated glomerular filtration rate (eGFR) are a biomarker of chronic kidney disease. Here, the authors reinterpret the genetic architecture of kidney function across ancestries, to identify not only genes, but the tissue and anatomical contexts of renal homeostasis.

## Introduction

Chronic kidney disease (CKD), defined by an estimated glomerular filtration rate (eGFR) lower than 60 ml/min/1.73 m^2^ for three or more months^1^, is a global health concern and is associated with premature death^2,3^. CKD has a prevalence of 15% and affects 30 million people in the US^4,5^. In addition to the risk of progressing to end-stage-renal disease (ESRD), CKD is associated with significant cardiovascular morbidity and mortality^6,7^. Patients with an eGFR of <15 ml/min/1.73 m^2^, for example, have a threefold increased mortality than those with normal renal function^8^.

Diabetes is the most common comorbidity associated with ESRD worldwide, occurring in 44–60% of ESRD cases^4,5^. CKD in diabetics is multifactorial and also related to hyperglycemia, hypertension, atherosclerosis, and aging. Currently there are few therapies that slow CKD progression and life-extending treatments for ESRD are restricted to dialysis and transplantation.

There is a great need to understand the biological mechanisms that lead to CKD so that treatments that target those biological factors can be developed. Kidney function, as measured by eGFR, is a heritable trait^9^ that has been studied in genetic association studies, and over 50 eGFR loci have been definitively identified^10–13^. A small proportion of variance in eGFR is explained by the subtle effects of previously reported variants, and the genetic etiology of this trait is highly complex. The inferences of what genes influence eGFR are often unclear from loci detected by genome-wide association studies (GWAS) of common variants, which comprise a large proportion of known genetic determinants. Regulatory effects may account for significant additional heritability in GWAS, and GWAS results are enriched for regulatory single-nucleotide polymorphisms (SNPs) compared with the proportion of the genome containing regulatory elements^14–16^. In recent large-scale GWAS meta-analyses of eGFR, it was shown that many significant SNPs map into tissue-specific regulatory regions, and that gene expression may mediate many of the associations between genetic variants and eGFR^10,12^. Several methods were recently developed to leverage multiple variants to perform gene-based tests of association between imputed gene expression levels and phenotypes^17–20^. These tests are tissue-specific and provide effect estimates with interpretable direction and magnitude compared with studies that only evaluate associations between SNPs and traits.

Thus, to capitalize on our large electronic health record-based study of patients receiving care in a standardized setting and to leverage available analytical possibilities, we conduct a transethnic GWAS of eGFR in 280,722 participants of the U.S. Veteran’s Administration Million Veteran Program, with replication in an additional 765,289 participants from the Chronic Kidney Disease Genetics (CKDGen) Consortium. In addition, because of the important role of diabetes in nephropathy, we stratify analysis by diabetes status. We also evaluate associations between genetically predicted gene expression (GPGE) in a human healthy kidney expression quantitative trait locus (eQTL) reference panel^21^ and eGFR, followed by comparison of significant GPGE associations with gene expression profiles in murine kidney cell types^22^ to identify the specific cells where gene expression effects likely arise. Finally, we evaluate clinical translation by performing a phenome-wide association study (PheWAS)^23^ of a weighted genetic risk score (GRS) of eGFR in the electronic health records across 192,868 MVP participants.

## Results

### MVP characteristics

A total of 280,722 participants were available from MVP for analyses of eGFR, ~33% were diabetic (Table 1). Most participants were non-Hispanic whites (80%), male (93%), and hypertensive (70%). When evaluating the data stratified by diabetes status, there were more diabetics than non-diabetics who were hypertensive within both race groups (non-Hispanic white diabetics and hypertensive 91%; non-Hispanic black diabetics and hypertensive 93%). Across both race groups, eGFR was lower in diabetics than in non-diabetics, and eGFR was higher in non-Hispanic blacks than in non-Hispanic whites.

**Table 1:** Characteristics of Million Veteran Program discovery sample

Characteristic	Overall	Non-Hispanic white diabetic (*n* = 70,762)	Non-Hispanic white non-diabetic (*n* = 152,624)	Non-Hispanic black diabetic (*n* = 20,967)	Non-Hispanic black non-diabetic (*n* = 36,369)
Hypertension (*n*, [% Yes])	198,137 (71%)	64,493 (91%)	90,875 (59%)	19,459 (93%)	23,310 (64%)
Gender (*n*, [% Female])	20,733 (7.4%)	3081 (4.4%)	12,986 (8.5%)	1781 (8.5%)	2885 (16%)
Age (mean [SD], years)	62.8 (13.2)	67.4 (9.8)	62.5 (14.3)	62.1 (9.9)	55.4 (12.5)
BMI (mean [SD], kg/m^2^)	30.1 (6.0)	32.1 (6.4)	29.0 (5.4)	31.9 (6.5)	29.4 (5.9)
eGFR (mean [SD], mL/min/1.73 m^2^)	79.1 (20.7)	72.3 (20.6)	79.0 (18.8)	80.4 (24.6)	89.4 (21.6)

*SD* standard deviation, *BMI* body mass index, *kg* kilograms, *m*^2^ meters squared

### Transethnic GWAS

Meta-analyses of GWAS data were performed on all variants (minor allele frequency, MAF, ≥1%), with data stratified by race, diabetes status, and hypertension status. Meta-analysis of both race groups across all diabetes and hypertension strata identified 136 genome-wide significant loci, of which 122 were available and evaluated for replication in a transethnic GWAS meta-analysis of eGFR from the CKDGen Consortium (*n* = 765,289). Fourteen of these SNPs or proxies from the all-ancestry analyses were unavailable for replication.

**Fig. 1:**
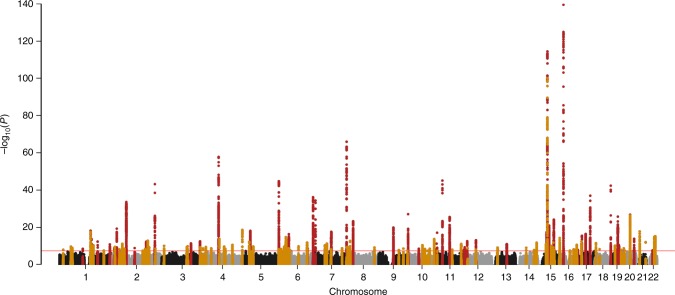
Manhattan plot summarizing transethnic discovery meta-analysis of eGFR. The *y* axis shows the –log10 *P*-values and the *x* axis shows the chromosomal positions. The horizontal red line represents the thresholds of *P*-value = 5 × 10^−8^ for genome-wide significance. SNPs in red are in previously-identified kidney function loci, whereas SNPs in orange are in novel loci

Of the 122 variants, 79 were replicated at the Tier 1 definition (*p* ≤ 5 × 10^−8^) and an additional 28 were Tier 2 replicated (5 × 10^−8^ < *p* ≤ Bonferroni correction for 122 SNPs = 0.0004), and 11 were Tier 3 replicated (0.0004 < *p* ≤ 0.05) (Supplementary Data 1, Table 2, Fig. 1). Only four showed no evidence of association in CKDGen. Meta-analysis of *p*-values from MVP and CKDGen results identified all Tier 1 and seven Tier 2 results as genome-wide significant. Across the three tiers of successful replication, 2.75% of variance in eGFR was explained. Fifty-seven of these results have been previously detected in GWAS of eGFR, while six others have associated with overt kidney disease, microalbuminuria, or other related phenotypes in prior studies. Among 64 novel variants, the most significantly associated was rs2823139 near *NRIP1* (*p*_discovery _= 1.82 × 10^−18^, effect = -0.45 mL/min/1.73 m^2^ [standard error (SE) = 0.051]; *p*_replication_ =  5.24 × 10^−16^). Evaluation of conditionally independent signals within the discovery meta-analysis results using GCTA^24^ identified 18 SNPs from 15 loci (five novel) that are significantly independently associated with eGFR (Supplementary Table 1).

**Table 2:** Summary of significant known and novel loci from analysis of common variants

	Number of loci	Average effect^a^ (SD)	*P*-values*
**Known loci**	54	0.49 (0.28)	0.0025
**Novel loci**	64	0.38 (0.17)	
**Tier 1**	79	0.45 (0.26)	
**Tier 2**	28	0.41 (0.19)	
**Tier 3**	11	0.36 (0.10)^b^	
**Diabetics**	35	0.90 (0.61)	0.001
**Non-diabetics**	98	0.52 (0.28)	

Tier 1 = First tier significance criteria: GWAS significance at discovery + replicationpassing Bonferroni threshold + consistent directions of associations between discovery and replication sets

Tier 2 = Second tier significance criteria: GWAS significance at discovery + replication *p* between 5 × 10^−8^ and 0.00040004 + consistent directions of associations between discovery and replication sets

Tier 3 = Third tier significance criteria: GWAS significance at discovery + replication *p* between 0.0004 and 0.05 + consistent directions of associations between discovery and replication sets

*P-value from two-sample *t*-test comparing mean effect estimates

^a^Average effect = average and standard deviation of the absolute value of beta-estimates from MVP, in mL/min/1.73 m^2^

^b^Excluding two extreme outliers

We also conducted analyses limiting to non-Hispanic white individuals. Similar to the trans-ancestry analyses, the majority of variants replicated in CKDGen (*n* = 567,401; Supplementary Data 2). Six variants failed to replicate (*p* > 0.05). Of the 105 replicated SNPs, 61 were Tier 1 (*p* ≤ 5 × 10^−8^), 29 were Tier 2 (5 × 10^−8^ < *p* ≤ 0.00048), and 9 were Tier 3 (0.00048 < *p* ≤ 0.05).

We identified 14 variants that were significant in non-Hispanic blacks (Supplementary Table 2) within our MVP discovery population. Among these four were novel (rs75113983 near *OLFR690*, rs200950799, which is intergenic on chromosome 12, rs144803907 near *C15orf43*, rs10084572 near *AGPAT3*). Only one of these novel variants was also identified in non-Hispanic white analyses (rs13230509). An interesting finding among the non-Hispanic blacks is the strong association with rs334 (*p* = 1.54 × 10^−18^) in *HBB*, the variant which encodes the sickle cell allele of beta globin. The origins of this variant have been recently described^25^. The derived A allele on the + strand that encodes for the sickle version of the beta globin protein is associated with lower kidney function. This allele has also been previously associated with increased urinary albumin-to-creatinine ratio^26^.

We compared the effect sizes for significant discovery GWAS results across non-Hispanic black and non-Hispanic white subjects MVP (Supplementary Fig. 1, Supplementary Table 3). Most of the effects were in the same direction except for three variants. One was a novel variant (rs142314590 in *LDB2*) and two were known variants (rs532086 and rs2235826 near *C2* and *PRK1*, respectively). The effect sizes across non-Hispanic black and non-Hispanic white subjects were more correlated among known (*r*^2 ^= 0.25) loci than novel (*r*^2^ = 0.13) variants (Supplementary Table 3). The overall allele frequencies across ancestry groups for significant variants were highly correlated (*r*^2^ = 0.67).

### Diabetes stratified GWAS

There were 91,279 patients with diabetes. In our GWAS for this group we identified 32 variants reaching genome-wide significance, which make up a subset of the loci discovered in combined analysis. Seventeen of these variants were in known loci and 15 were novel loci (Supplementary Data 3, Supplementary Figs. 2 and 3). The top six hits were near *UMOD* (*p* = 2.43 × 10^−82^), *PRKAG2* (*p* = 7.89 × 10^−23^), *MPPED2/DCDC5* (*p* = 9.85 × 10^−22^) *GATM*/*SPATA5L1* (*p* = 2.21 × 10^−17^), *SHROOM3* (*p* = 1.63 × 10^−13^), and *HBB* (*p* = 7.95 × 10^−13^). Comparison of the association effect sizes in the stratified analysis in subjects with (Supplementary Data 3) and without diabetes (Supplementary Data 4) demonstrated generally consistent effects reflecting the shared pathways between diabetic and non-diabetic kidney disease (Supplementary Fig. 4).

### LD score regression

Subsequently, we utilized the LD Score Regression approach^27^ in each contributing group to ascertain whether inflation was due to residual population stratification or polygenicity. Calculation of the intercept in the MVP non-Hispanic white discovery analysis datasets were 1.02 (SE = 0.01), 1.04 (SE = 0.01), 1.03 (SE = 0.01), and 0.99 (SE = 0.01), for diabetics with hypertension, non-diabetics with hypertension, non-diabetics without hypertension and diabetics without hypertension, respectively, suggesting that little of the observed inflation in the lambda is due to population stratification (Supplementary Table 4). Similarly, intercepts in the MVP non-Hispanic black discovery analysis datasets were 1.01 (SE = 0.003), 1.02 (SE = 0.004), 1.00 (SE = 0.003), and 1.00 (SE = 0.003), for diabetics with hypertension, non-diabetics with hypertension, non-diabetics without hypertension, and diabetics without hypertension, respectively.

### eGFR heritability estimation

Evaluation of SNP-based heritability was assessed using LD score regression across groups and indicated that eGFR was most heritable among non-diabetics and non-hypertensive individuals in both non-Hispanic whites (*h*^2^ = 0.15, SE = 0.02) and non-Hispanic blacks (*h*^2^ = 0.13, SE = 0.04) (Supplementary Table 4). In general, heritability was higher in non-Hispanic whites than in non-Hispanic blacks.

### Human kidney genetically predicted gene expression (GPGE)

**Fig. 2:**
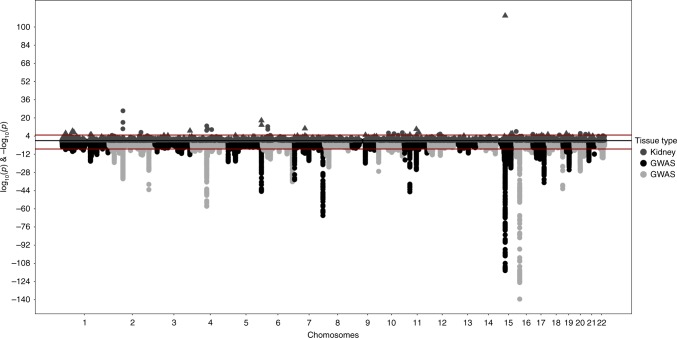
Juxtaposed mirror plots for S-PrediXcan and GWAS for eGFR. Log_10_(*p*-values) for associations between genetically predicted gene expression (GPGE) analyses with eGFR in kidney tissue are juxtaposed with –log_10_(*p*-values) from transethnic GWAS analyses (triangles represent odd numbered chromosomes, circles represent even numbered chromosomes). GWAS plot represents transethnic discovery meta-analysis results (chromosomes alternate by light gray and black coloration)

Common variants from transethnic meta-analyses were used to evaluate the association between eGFR and GPGE in human healthy kidney tissue using S-PrediXcan^17^ with the healthy kidney eQTL reference described by Ko et al.^21^ (Fig. 2, Supplementary Data 5). We identified 45 significant results for transethnic analyses, among these 36 were novel genes which had not been identified by GWAS, either in the original reports or by the GWAS catalog mapping. The strongest result was with a known CKD locus, *SPATA5L1* (effect = −2.38; *p* = 1.01 × 10^−110^). The strongest novel result was at *TPRKB*, a protein coding gene for the TP53RK-binding protein (effect = −10.78; *p* = 3.40 × 10^−22^). Twenty-five of the 45 genes with significant results were associated with a decrease in kidney function with increasing renal gene expression. Among all significant results, 19 also had SNPs likely to be causal for both gene expression and eGFR differences, as identified through the COLOC approach.

We also conducted secondary GPGE analyses stratifying subjects by diabetes status (Supplementary Data 5). *SPATA5L1* was the strongest result in analyses of diabetic and non-diabetic participants, with stronger effects estimated in the non-diabetics. In analyses of diabetic participants, we identified seven significant associations. Two of the genes (*HLA-H*, *UBD*) identified in analyses of diabetic participants were not observed in overall analyses (combining diabetics and non-diabetics) or in analyses limiting to non-diabetic subjects. Both genes were associated with a decrease in kidney function with increasing expression and neither gene has been previously associated with eGFR. *HLA-H* has been associated with non-albumin protein levels and *UBD* with blood proteins^28,29^. All significant results in analyses of non-diabetics were also identified in overall analyses combining diabetics and non-diabetics.

### Evaluation of GPGE results in murine kidney cells

**Fig. 3:**
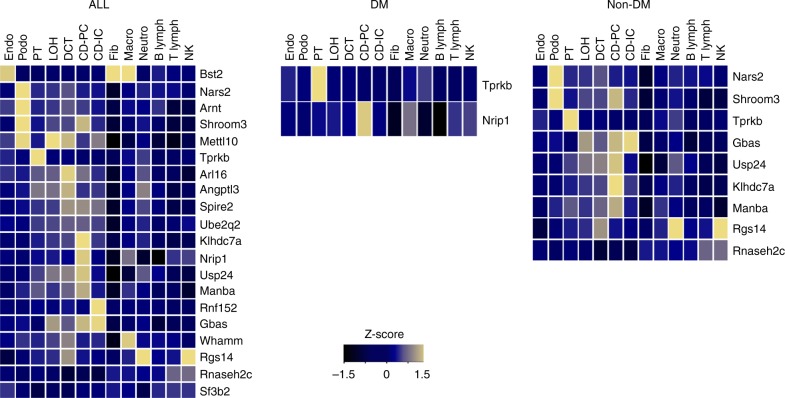
Mapping eGFR-associated genes to kidney cell type clusters. Average expression level of GWAS/eQTL defined genes across overall transethnic (ALL), diabetic (DM), and non-diabetic (Non DM) analyses. Mean expression values of the genes were calculated in each cluster. Color scheme is based on *z*-score distribution. Each row represents one gene and each column is single cell type cluster (as defined by Park et al.^22^) on the heatmap. Endo: endothelial, vascular, descending loop of Henle, Podo: podocyte, PT: proximal tubule, LOH: ascending loop of Henle, DCT: distal convoluted tubule, CD-PC: collecting duct principal cell, CD-IC: CD intercalated cell, Fib: fibroblast, Macro: macrophage, Neutro: neutrophil, NK: natural killer cell

We also evaluated homologs of genes identified in GPGE analyses using an atlas of kidney cell type-specific RNA expression from single cell sequencing of murine kidney cells^22^ (Fig. 3, Supplementary Table 5). Cells were clustered into 13 types that represent structural features and other cell types of the kidney. We identified six genes (*NARS2*, *ARNT*, *TPRKB*, *RNF152*, *BST2*, and *RGS14*) across five cell types (podocyte, proximal tubule, collecting duct principal cell, fibroblast, and neutrophil) that had a 1.96 or greater fold increase in gene expression, though none of these were significant after accounting for multiple tests. Cross-referencing protein expression levels in the Human Protein Atlas confirmed findings from murine kidney, including higher expression of TPRKB protein in tubules compared with glomeruli (Supplementary Table 6).

### eGFR risk score PheWAS

To assess the potential pleiotropic effects of associated eGFR variants we tested the association between an eGFR weighted GRS (w-GRS) and diseases throughout the phenome using 63 SNPs with independent weights identified previously in the CKDGen Consortium^10,30^ (Supplementary Table 7) and clinical phenotype data from 192,868 self-reported/administratively identified non-Hispanic white MVP individuals (Supplementary Table 8). We regressed PheWAS outcomes onto the w-GRS, adjusting for sex and the top ten principal components of ancestry. We identified nine conditions that were significantly associated with the eGFR w-GRS. The majority were diseases related to genitourinary systems (*n* = 6), the strongest result was with chronic renal failure (*p* = 3.55 × 10^−57^, OR per SD of w-GRS = 0.88). Interesting results included calculus of kidney, calculus of ureter, and urinary calculus, which were all significant and positively associated with the weighted GRS (i.e., increased eGFR [improved kidney function] associated with increased risk of kidney stones). In addition, we observed significant associations with stage III of CKD and renal failure.

## Discussion

We present the results from a multi-omic transethnic GWAS of eGFR with discovery and replication in over a million participants. We identified several novel loci using multiple statistical and bioinformatics approaches and validated previously reported loci, with replicated SNPs explaining 2.75% of eGFR variance. Significant strengths of this study include our large discovery and replication populations, transethnic analyses of diverse populations, incorporation of GPGE from a healthy human kidney reference, identification of cell-specific eGFR-associated gene expression from murine kidney, and evaluation of the clinical phenome through PheWAS of an eGFR w-GRS. We also assessed heterogeneity of effect estimates at GWAS loci between diabetic and non-diabetic populations. In addition to identifying novel and known loci, our discovery analyses using the MVP population showed heritability estimates consistent with published studies of eGFR, supporting the quality of our EHR phenotyping^30^. These data provide insight into the genetic architecture and clinical factors of eGFR.

The limitations of estimating GFR from creatinine and/or cystatin C include low sensitivity in detecting early CKD and poor prediction of the course of CKD. These limitations have been described previously in detail^31–33^. However, despite these limitations, eGFR is the outcome most often used in genetic association studies of kidney function because of its clinical utility and the translational potential of inferences, as well as the availability of large numbers relative to alternative measures.

We identified several novel common variants associated with eGFR, some of which may tag genes implicated in Mendelian forms of kidney disease. For instance, we detected a common (and reportedly benign) missense variant in *PKD1* (polycystin 1) associated with eGFR. Mutations in *PKD1* can cause autosomal dominant polycystic kidney disease (OMIM #173900). Variants in *NOS3* (nitric oxide synthase 3), where we also detected an association with eGFR, may act as a modifier among those with polycystic kidney disease^34^ through the nitric oxide pathway. We also identified associations near the *NRIP1* gene (nuclear receptor interacting protein 1), with supporting GPGE evidence. *NRIP1* mutations have recently been implicated in congenital anomalies of the kidney and urinary tract (OMIM# 610805), which are a leading cause of CKD among those under 30^35^. It has been suggested that mutations in this gene cause CKD through disruption of retinoic acid signaling.

Novel SNP associations were found at or nearby genes previously reported in GWAS of other renal and urological phenotypes. Specifically, loci were identified with urate/urea/uric acid levels (*KLHDC7A*,^36^ *MTX1,*^37^ *RREB1*,^38^ and *MIR1538*^28,38^), urine albumin-to-creatinine ratio (*C9orf3*^39^), IgA Nephropathy (*MTMR3*^40^), and frequency of urinary tract infection (*ZNF165*^41^). We also observed an association at a pharmacogenetic locus strongly related to tacrolimus dose in renal transplant patients^42^, as well as development of new-onset diabetes after transplant^42^. However, transplant patients were excluded from this analysis.

Loci implicated in platelet and red blood cell phenotypes were also represented among novel eGFR-associated SNPs, including *TET2*, *HIST1H1C*, *UBE2H*, *TRIB1-LOC105375746*, *FAM53B*, *TPM1*, *NRIP1*, *A4GALT*, *DOCK7*, *PLCB1*, and *SERTAD2*^28,43^. It has been previously shown that mean platelet volume, platelet counts, and platelet distribution width are associated with eGFR^44,45^. Higher values of mean platelet volume are observed among CKD patients^46^, while platelet count and distribution width decrease along with eGFR. CKD patients also demonstrate an attenuated response to antiplatelet therapy relative to those without renal insufficiency^47^.

Ischemic stroke is a common secondary occurrence in CKD patients, which may be due to shared risk factors^48^. Reduced kidney function among stroke patients is a risk factor for mortality^49,50^. We identified four novel eGFR-associated loci which have been previously associated with ischemic stroke in other studies, specifically *USP38*, *ZFHX3*, *PMF1-BGLAP*, and *TTBK1*^51–53^. A previous analysis of the *APOL1* high risk variants showed increased risk of ischemic stroke^54^. Combined with our results, this suggests that the genetic architecture of kidney function may predispose to other small vessel disease outcomes.

We examined the *APOL1* locus extensively for association with eGFR in black MVP participants in anticipation that there would be a relationship there with eGFR and did not observe an association with an additive genotype coding. It has been suggested in multiple studies where participants had predominantly normal kidney function that *APOL1* is a kidney disease gene and has not been significantly associated with eGFR^55–57^. This suggests that the lack of association between *APOL1* genetic variants and eGFR is likely not due to low power, as has been true in previous studies of African-descent populations.

We evaluated our transethnic sentinel SNPs for consistency of effect size across racial groups and found that in general effects were of similar direction, with only three variants being of opposite effect in non-Hispanic blacks (Supplementary Fig. 1). These included two known variants: rs532086 at *C2*, rs2235826 at *PCK2* and a novel variant, rs12646572 at *LDB2*. Effect sizes of these three SNPs in non-Hispanic blacks were small (<0.01) and likely represented null effects. Comparison of variants identified in non-Hispanic blacks with non-Hispanic whites demonstrated consistency of direction across known variants with effect sizes generally similar or larger in non-Hispanic blacks. When eGFR effects across the entire genome were compared between white and black MVP participants using the Popcorn software package^58^, the genetic effects were highly correlated and not significantly different from no difference (*r*_*ge*_ = 0.94, *p* = 0.45). Novel variants detected in non-Hispanic blacks were not observed in non-Hispanic whites. Our analysis of eGFR in non-Hispanic whites was largely consistent with previous European-ancestry cohorts, replicating many strongly associated loci among our top hits, such as *UMOD*, *GATM*/*SPATA5L1*, *SHROOM3*, *CPS1*, *PRKAG2*, and *SLC31A1*^10,11,59^.

An interesting finding among the non-Hispanic blacks is the strong association with rs334 (*p* = 1.54 × 10^−18^, SNPTEST info score = 0.75) in *HBB*, which encodes the sickle cell allele of beta globin. This variant has been previously associated with increased urinary albumin-to-creatinine ratio^26^. The sickling allele was present at a frequency of 5.7% among MVP non-Hispanic blacks, despite sickle cell trait carriers being restricted from serving in certain roles in the United States armed forces^60^. This observed frequency is higher than that in the African Americans from the southwestern United States in 1000 Genomes (MAF = 1.6%), but substantially lower than in continental African populations (MAF 10.1–13.9%). Screening for sickle cell in the United States Military has undergone several changes since the initial policy was introduced in 1969, in which the Navy began screening all recruits^60,61^. Currently universal sickle cell screening is performed prior to military ascension in the U.S. Navy, Air Force, and Marine Corps, while in the U.S. Army screening is performed only in specific scenarios related to deployment and certain occupational hazards^62,63^. Similarly, in other countries’ militaries, sickle cell allele carriers may be barred from diving, submarine, and aircrew service^64^. A recent study found that sickle cell trait was not associated with increased risk of mortality among black soldiers in the U.S. Army, but that it was associated with increased risk of exertional rhabdomyolysis (breakdown of skeletal-muscle tissue)^65^. However, the effect of sickle cell trait was less than that of recent statin use. Our results suggest that this allele also negatively affects kidney function, which may indicate that carriers should be monitored clinically for decreases in eGFR.

Associations were also identified near loci previously implicated in diabetes and insulin/glucose homeostasis (*CDKAL1*, *SLC9B2*, *RREB1*, *RAI1*, and *PPARG*)^28,66–68^. *CDKAL1*, *RREB1,* and *PPARG*  were not significant in either arm of the diabetes stratified analysis, while *RAI1* and *SLC9B2* were significant in the non-diabetes group. We identified 36 SNPs that reached genome-wide significance in our diabetes strata, 12 were novel and not significant in non-diabetics, but were observed in the overall transethnic analysis combining diabetic groups. We also did an exploratory comparison of the association effect size in the subjects with and without diabetes. The effects were generally consistent across groups (Supplementary Fig. 3). Uromodulin (*UMOD*) has been recently detected in a GWAS of diabetic eGFR that limited to diabetic subjects^69^. We observed similar associations for *UMOD* in separate analyses within diabetic and non-diabetic participants (sentinel SNP [A effect allele] effect size for *UMOD* diabetic 0.11 and non-diabetic 0.07), suggesting the association is not specific to diabetics. *UMOD* is known to affect kidney function and mutations in that gene cause several syndromic kidney disorders. Few variants have reached genome wide significance for studies of eGFR in patients with diabetes, potentially due to power and heterogeneity of renal disease phenotypes in diabetics^70,71^.

We leveraged the human healthy kidney eQTL reference first described by Ko et al.^21^ to investigate the relationship between GPGE and eGFR using S-PrediXcan. We detected associations with 45 genes, 18 of which are not annotated for any trait in the GWAS catalog, either by the original report or by the catalog mapping. When we restricted the GWAS catalog to genes identified by studies of eGFR, CKD, kidney disease, urinary albumin to creatinine ratio, or urinary metabolites, 36 of the 45 genes we detected were novel. The most statistically significant association with GPGE was for the spermatogenesis associated protein 5-like 1 gene (*SPATA5L1*). This locus has been previously reported to be associated with eGFR^10,72^. However, there are several genes in the region and Köttgen et al^72^ suggested the nearby gene glycine amidinotransferase (*GATM*), based on a pathway-level connection of that gene to creatinine biosynthesis. The sentinel non-coding SNP rs2467853 in this region is within the *SPATA5L1* gene and is more strongly associated with *SPATA5L1* expression in human kidney than *GATM* (rs2467853 *SPATA5L1* beta = 0.77, *p* = 8.2 × 10^−8^; *GATM* beta = 2 × 10^−4^, *p* = 0.99)^73^. In our evaluation of RNAseq and genotypes in *cis* with *GATM* from healthy human kidney, no models could be constructed that sufficiently predict *GATM* expression to use in GPGE analysis. We detected an association between GPGE and eGFR for *SPATA5L1* and not for *GATM*, as well as a COLOC signal with a high posterior probability (P4 = 0.98) of several SNPs in the region being causal for both *SPATA5L1* expression levels and the eGFR association signal, and no COLOC or GPGE evidence supporting *GATM*. The mechanism underlying the relationship between *SPATA5L1* gene product abundance and kidney function is unclear.

The most statistically significant novel gene detected in S-PrediXcan analysis was the TP53RK-binding protein (*TPRKB*), which is significantly highly expressed in murine proximal tubules. Consanguineous families with homozygous missense mutations in *TPRKB* have been diagnosed with Galloway-Mowat syndrome-5 (OMIM #617731), in which affected people exhibit early-onset nephrotic syndrome, as well as dysmorphologies and delayed psychomotor development^74^. The *NARS2* gene is also novel and significantly highly expressed in murine podocytes. Mutations of this gene have been observed in a phenotype similar to Alpers syndrome that included renal dysfunction (OMIM #612803)^75^.

It is also notable that *UMOD* from the chr16p12.3 region was not associated in the GPGE association analysis. No SNPs in the *UMOD* region were sufficiently associated with *UMOD* expression in the human kidney reference data to create predictive models for S-PrediXcan analysis. This suggests that the relationship between *UMOD* genotypes and kidney function is mediated by a mechanism other than gene expression, or that the proportion of cells from which *UMOD* is expressed is small and difficult to detect in bulk RNA sequencing of whole kidney. The sentinel SNP nearby *UMOD* from our study (rs77924615) is a nominally significant eQTL within NephQTL^76^ (a database of nephrotic syndrome human kidneys) in the kidney tubules (*p* = 0.0014) and has a non-significant effect in the glomerulus (*p* = 0.079). The conditionally independently associated SNP rs111285796 in *UMOD* is also nominally associated in NephQTL with *UMOD* expression in tubules (*p* = 3.6 × 10^−4^) and less associated with other genes at this locus (*PDILT p* = 0.047), and less significant for glomerulus (*UMOD p* = 0.021). Within a healthy kidney eQTL atlas^77^ (Online Methods) rs111285796 is not associated with UMOD in tubules (*p* = 0.214) but is nominally associated in glomerulus (*p* = 0.004). This demonstrates that there are important differences between the nephrotic and healthy kidney reference data with regard to *cis*-eQTL effects at this locus and suggests that SNPs in this locus influence gene expression in a context-dependent manner.

We constructed a w-GRS of eGFR and performed a PheWAS using EHRs from 192,868 non-Hispanic white MVP participants. To our knowledge this is the first PheWAS of an eGFR w-GRS. We observed several associations between our w-GRS and kidney disease-related phenotypes. The most significant PheWAS association (*p* = 3.55 × 10^−57^) was with chronic renal failure, followed by related renal failure phenotypes, and CKD stage III (*p* = 6.13 × 10^−24^), all diseases defined by eGFR. Our w-GRS was associated with hypertensive kidney disease but not essential hypertension. In addition, we observed an association with kidney stones that was positively associated with our w-GRS; a relationship that has not been previously reported with eGFR. Our results are consistent with a prior study that reported an association between increased eGFR and hypercalciuria^78^. It is known that some individual genes that influence eGFR are also important for kidney stone formation. For example, decreased production of *UMOD* is associated with kidney stone formation as uromodulin impairs the aggregation of calcium oxalate crystals^79^, hypertension, arterial stiffness, and CKD.

In conclusion, we identified multiple novel loci associated with eGFR levels after conducting a transethnic GWAS and several post-GWAS bioinformatic analyses. We identified several novel loci and genes with additional confirmation and refinement through GPGE analyses, including cell-specific expression. We observed consistent effects across racial groups for associated GWAS loci. Furthermore, we evaluated the clinical phenome associated with our eGFR w-GRS and identified associations among diseases related to kidney and endocrine disease phenotypes. Overall, our study leveraged a racially diverse clinical population to identify novel eGFR loci common across racial groups and remapped previously reported loci using GPGE, leading to a greater understanding of the genetic architecture of kidney function.

## Methods

### Ethics statement

The central Veterans Affairs (VA) institutional review board (IRB) and site-specific IRBs approved the Million Veteran Program study.

### The Million Veteran Program

The Million Veteran Program (MVP) is a large cohort of fully consented participants who were recruited from the patient populations of 63 VA medical facilities. MVP recruitment was initiated in 2011 and conducted in-person, after responding to an invitation letter. Full MVP participation includes completion of baseline and lifestyle surveys, providing access to medical records, a blood sample, and giving permission to recontact. Informed consent is provided after counseling by research staff and access to informational materials. All study materials and protocols are approved by the VA Central Institutional Review Board. Genotyping was performed on the Affymetrix Axiom Biobank Array chip, with custom content included to provide better coverage of African and Hispanic haplotypes. All samples are de-identified for research purposes, and investigators are not permitted or able to link study data to a participant’s identity.

Blood samples were obtained from MVP participants by phlebotomists and shipped to a central biorepository in Boston, Massachusetts for biobanking. DNA was extracted and provided to two external sites for genotyping. Standardized quality control and genotype calling algorithms using the Affymetrix Power Tools Suite (v1.18) were applied to the data in batches by the MVP genomics working group. Quality control pipelines included the exclusion of duplicate samples, those with discordant reported and genotyped sex, and samples with more heterozygosity than expected. One of each pair of related individuals as measured by the KING software^80^(halfway between 2nd and 3rd degree relatives or closer) were excluded from genetic analysis.

 Prior to imputation, variants that were poorly called or that deviated from their expected allele frequency based on reference data from the 1000 Genomes Project^81^ were excluded. After pre-phasing using EAGLE v2^82^, genotypes from the 1000 Genomes Project phase 3, version 5 reference panel were imputed into Million Veteran Program (MVP) participants via Minimac3 software^83^. Principal component analysis was performed using the FlashPCA^84^, to generate the top ten genetic principal components explaining the greatest variability.

Information on race and ethnicity (Hispanic: Yes or No) were extracted from standardized survey forms (self-report), or from the corporate data warehouse (CDW), or observational medical outcomes partnership (OMOP) data, when information from self-report was unavailable, and data were combined to form administratively assigned variables. Race and ethnicity categories used in this study included non-Hispanic whites and non-Hispanic blacks.

Baseline estimated glomerular filtration rate (eGFR) was determined using the creatinine closest to enrollment. For the vast majority of the patients creatinine was measured using the IDMS reference method. GFR was calculated using chronic kidney disease epidemiology collaboration (CKD-EPI) serum creatinine equation^85^. The eGFR CKD-EPI equation is (1):

GFR = 141 ×min(Scr ×*κ*^−1^,1)^*α*^ × max(Scr × *κ*^−1^, 1)^−1.209^ × 0.993^Age^  × 1.018 [if female] × 1.159 [if Black]

where Scr is serum creatinine (mg/dL), *κ* is 0.7 for females and 0.9 for males, *α* is −0.329 for females and −0.411 for males, min indicates the minimum of Scr × *κ*^−1^ or 1, and max indicates the maximum of Scr × *κ*^−1^ or 1. We excluded individuals that were on dialysis, had a kidney transplant, amputees, individuals on HIV medications which may increase creatinine clearance, BMI < 18, and Scr values less than 0.4 mg/dl as they may have represented lab errors. Diabetic patients were defined as those subjects on any anti-diabetic medications or those who had at least two outpatient ICD-9 codes for diabetes (ICD9 250.*) on separate dates within 365 days prior to enrollment. Subjects lacking codes and not on anti-diabetes medications were categorized as non-diabetics. Hypertension was defined as the presence of a hypertension code, being on antihypertensive drug or having two SBP’s > 140 mmHg and/or two DBP’s > 90. BMI was estimated using the closest weight to the GFR measure, and the height mode as weight (kg) × (height (m))^−2^.

### MVP GWAS analysis

For the MVP GWAS we performed linear regression association tests with additive models for untransformed eGFR. We adjusted linear regression models analyzing SNP associations for age at eGFR measure, age^2^, sex, BMI, and the top ten genetic principal components (PCs) in analyses. All primary analyses for the MVP were conducted by stratum of administratively assigned race, as well as by the presence or absence of diabetes and hypertension. All regression-based analyses were conducted in SNPTEST-v2.5.4-beta^86^. Inference was limited to genotyped and imputed variants with SNPTEST Info scores of 0.4 or higher, with Hardy-Weinberg equilibrium *p*-value > 5 × 10^−8^ for common variant analysis (MAF > 0.1). Meta-analyses across race and strata were performed using fixed-effects, inverse variance-weighted meta-analysis implemented in METAL^87^.

Genomic inflation factors were calculated, and *λ*_*GC*_ for the discovery from MVP were 1.11, 1.16, 1.15, and 1.01 for the diabetic hypertensive participants, non-diabetic hypertensive participants, non-diabetic non-hypertensive participants, and diabetic non-hypertensive participants in whites, respectively, 1.03, 1.03, 1.02, and 0.99 for the diabetic hypertensive participants, non-diabetic hypertensive participants, non-diabetic non-hypertensive participants, and diabetic non-hypertensive participants in blacks, respectively, and 1.36 in the overall discovery analysis (Supplementary Fig. 3).

Genome-wide significant loci were defined by one or more SNPs attaining genome-wide significance (*p* < 5 × 10^−8^) which were at least 1MB away from other signals, or if within that distance, were not in linkage disequilibrium (*r*^2^ < 0.1). Sentinel variants and up to two proxies (*r*^2^ >  0.8 and prioritized by correlation and then by distance from lead SNP) were selected for replication.

We approximated the proportion of variance explained in the transethnic meta-analysis by all independent sentinel SNPs (novel and known) and novel SNPs, separately. Variance explained by each SNP was first estimated by the following equation (2):

$$r^2 = \chi ^2n^{ - 1}.$$

The sum of the variances of the independent sentinel SNPs for common variants provided estimates for the proportion of variance explained for all SNPs, and novel SNPs for eGFR. The transformation of the relationship between *t*-statistic and *r*^2^ to ${\mathrm{\chi }}^2$ statistic to *r*^2^ is described in Supplementary Note 1.

### CKDGen transethnic and European ancestry GWAS meta-analyses

The Chronic Kidney Disease Genetics (CKDGen) Consortium is a collaborative effort that includes mainly population-based studies from different ethnicities to perform GWAS of renal function traits aimed at uncovering the genetic basis of CKD. We interrogated data from the most recent CKDGen meta-analysis that included 121 GWAS, totaling 765,289 individuals of European (*n* = 567,401), East Asian (*n* = 165,726), South Asian (*n* = 13,359), African American (*n* = 13,842), and Hispanic (*n* = 4961) ancestries^88^ . Following a centralized analysis plan, participating studies estimated the eGFR based on serum creatinine using the Chronic Kidney Disease Epidemiology Collaboration (CKD-EPI)^85^ or the Schwartz’s^89^ equations, depending on whether adults or ≤18 year old children were concerned. eGFR values were winsorized at 15 and 200 ml/min/1.73 m^2^. Studies had a median mean eGFR of 89 ml/min/1.73 m^2^ (interquartile range: 81–94) and a median mean age of 54 years. Overall, 50% participants were females. Each study performed genotype imputation based on the Haplotype Reference Consortium (HRC) v1.1 or the 1000 Genomes Project phase 3 v5 ALL or phase 1 v3 ALL panels. Sex- and age-adjusted linear regression models were fitted to the logarithm of eGFR. GWAS were performed by regressing the residuals of the linear models on SNP dosage levels, assuming additive genetic effects. Family-based studies accounted for relatedness including kinship estimation or genetic principal components into the linear models. After selecting SNPs with imputation quality score >0.6 and minor allele count >10, genomic control correction was applied in case of an inflation factor *λ* > 1. GWAS were pooled using fixed effects inverse-variance weighted meta-analysis. After meta-analysis, SNPs that were not present in at least 50% of the studies were discarded, leaving 8,221,591 variants from ≥61 GWAS across all ancestries and 8,834,748 variants from ≥42 GWAS in the European ancestry subset. No further genomic control correction was applied. Genome-wide significance level was set at 5 × 10^−8^.

Summary statistics for selected variants from the CKDGen consortium were used for replication. 105 variants were assessed for the non-Hispanic white-only meta-analysis in a maximum of 567,453 individuals from up to 84 studies. As many as 120 studies provided summary statistics for 122 variants for a maximum of 765,346 participants for the all-ancestry analysis. We performed a sample-size weighted *z*-score linear combination meta-analysis of MVP and CKDgen results (Supplementary Data 1).

### DEPICT methods

Enrichment analyses in DEPICT^90^ were conducted using significant GWAS sentinel SNPs from three separate analyses as input: (1) transethnic analyses of all MVP subjects, (2) transethnic analyses of MVP subjects with DM, and (3) transethnic analyses of MVP subjects without DM. DEPICT incorporates predefined phenotypic gene sets from multiple databases with expression microarray data (Affymetrix HGU133a2.0) from more than37k subjects to provide gene sets with high expression for Medical Subject Heading (MeSH) tissue and cell type annotations. DEPICT output includes enrichment *p*-values for both tissue level and gene-set features, as well as an indicator for whether each enrichment test had an FDR *q*-value of <0.05.

### LD score regression and Popcorn

Subsequently, we utilized the LD Score Regression approach^27^ in each contributing group to ascertain whether inflation was due to residual population stratification or polygenicity. Among whites we used 1000 Genomes precomputed LD Scores and for non-Hispanic blacks we calculated LD Scores in 2217 African American participants from BioVU using Illumina Mega Array (Illumina, Inc) genotype data imputed to 1000 Genomes phase 3 haplotypes. Calculation of the intercept in the MVP non-Hispanic white discovery analysis datasets were 1.02 SE = 0.01), 1.04 (SE = 0.01), 1.03 (SE = 0.01), and 0.99 (SE = 0.01), for the diabetic hypertensive participants, non-diabetics hypertensive participants, non-diabetics non-hypertensive participants, and diabetic non-hypertensive participants, respectively, suggesting that little of the observed inflation in the lambda is due to population stratification. Similarly, intercepts in the MVP Blacks discovery analysis datasets were 1.01 (SE = 0.003), 1.02 (SE = 0.004), 1.00 (SE = 0.003), and 1.00 (SE = 0.003), for the diabetic hypertensive participants, non-diabetic hypertensive participants, non-diabetic non-hypertensive participants, and diabetic non-hypertensive participants, respectively. Heritability was also assessed within each strata of the MVP data using LD Score regression. We also used the related Popcorn^58^ software to evaluate the transethnic genetic effect correlation across the entire genome.

### Conditional analysis

For conditional analysis of common variants we used two parallel approaches implemented in the genome-wide complex traits analysis (GCTA) software:^24^ (i) genome-wide joint conditional analysis; and (ii) locus-specific conditional analysis.

(i)Genome-wide joint conditional analysis

Conditional analysis was conducted within GCTA software, using the –cojo method, which performs iterative conditional and joint analysis simultaneously with stepwise model selection^24^. The summary statistics from the GWAS discovery whites was used as the input summary data, and the imputed, hard-called BioVU EA genetic data (*n* = 19,726) was used as the reference genotype-level data, in PLINK format. Combination of these two input data files restricted the GCTA analysis to the imputed SNPs in common to the GWAS discovery meta-analysis (which was itself restricted to MAF > 1%). Within the BioVU genetic data, LD was calculated between all pairwise SNPs. A *p*-value cutoff of 5 × 10^−8^ was used as the selection threshold within GCTA, and the collinearity threshold was set at the default value of 0.9, so that SNPs are not selected if the multiple regression with the current SNPs in the model has *R*^2^ ≥ 0.9. After combining results across all 22 chromosomes, each trait-specific analysis resulted in a distinct set of jointly independent significant signals. Hence all final SNPs are pairwise-LD-independent.

(ii)Locus-specific conditional analysis

Analysis of each significant or previously reported locus was performed across all regional (1 Mb locus region centered ± 500 kb around the sentinel SNP) imputed SNPs with MAF ≥ 1%, conditioning on the sentinel SNP, using the --cojo-cond command in GCTA. As in the genome-wide approach, GWAS discovery meta-analysis results were used as the input summary data, with the BioVU EA imputed genetic data used as the reference PLINK dataset for LD computation. This approach provides conditional analysis results for each SNP within the implicated regions after conditioning on the sentinel SNP(s). These results yield a list of potential secondary SNPs which are evaluated according to the criteria below to identify those which are both significant and independent:

(a)*p* < 5 × 10^−8^ from original (unconditioned) GWAS discovery analysis, (i.e., SNP is significantly associated with eGFR itself)

(b)*p*_c_ < 5 × 10^−8^ from the conditional analysis (i.e., the SNP is significantly associated with eGFR after conditioning on the sentinel/published SNPs)

(c)independent of any of the sentinel SNPs (*r*^2^ <  0.1)

Significant independent SNPs meeting the above criteria, from any locuswere combined into a single list. This list is more comprehensive than that from approach (i), as it contains all possible secondary SNPs, rather than a single lead SNP at each independent signal. These secondary SNPs may be in LD with each other within a given locus.

The outputs from the two different approaches were then combined to identify those SNPs which are genome-wide significant in the discovery dataset and jointly independent on a genome-wide level. For robustness, a secondary signal was only claimed if the SNP is validated from both approaches.

### S-PrediXcan analysis

Genetically predicted gene expression was evaluated for the discovery GWAS with S-PrediXcan^9^, a gene-level approach which estimates the genetically determined component of gene expression in each tissue and tests it for association with SNP-level summary statistics. We utilized a human kidney gene expression atlas published by Ko et al.^21^ to conduct our genetically predicted gene expression analyses with our eGFR outcome.

### Human kidney compartment cis-eQTL analysis

Human kidney tissues (*n* = 151) were microdissected in RNAlater to obtain glomerular and tubular renal compartments. An unbiased review of the tissue section was performed by a renal pathologist by scoring of multiple parameters. One microgram of total RNA from each compartment was used for isolation of poly(A) purified mRNA using the Illumina TruSeq RNA Preparation Kit. Samples were sequenced in single-end 100 bp reads, and annotated RNA counts were calculated by Illumina’s CASAVA 1.8.2 to generate fastq. Alignment of trimmed reads to the Gencode human genome (GRCh37) was performed using STAR-2.4.1d^91,92^. RNA-seq data are available at Gene Expression Omnibus (GSE115098).

Compartmental eQTL data sets were generated from 121 tubule samples and 119 glomerulus samples, respectively^77^. The *cis* expression window was defined as 1 Mb on either side of the gene transcriptional start site. eQTL analyses were performed using linear regression in FastQTL software^93^ under an additive model and adjusted for six genetic PCs.

### Murine kidney single cell sequencing analysis

Homologs of human genes detected in S-PrediXcan analyses of healthy human kidney eQTL reference and GWAS summary statistics were further investigated for kidney cell type-specific RNA expression using single cell sequencing in murine kidney cells. Cells were clustered by expression profiles into groups representing kidney structural features and additional cell types found in the kidney^22^. Confirmation of murine kidney findings was performed through cross-reference of protein expression levels available in the Human Protein Atlas^94^.

### Genetic risk score construction

We constructed a weighted genetic risk score (w-GRS) for eGFR by calculating a linear combination of weights derived from previous publications from CKDGen^10,30^ for index SNPs at each of the 63 statistically significant replicated loci (Supplementary Table 7). GRSs were constructed for self-reported/administratively assigned non-Hispanic white individuals in the MVP only.

### Phenome wide association study analysis

We performed a phenome wide association study (PheWAS) of GRS in MVP non-Hispanic whites (*N*_max_ = 192,868), leveraging the full catalog of ICD-9 diagnosis codes. We used logistic regression to separately model each of 1813 PheWAS traits as a function of GRS, adjusted for sex and ten PCs. We report the results from these analyses as odds ratios where the estimate is the average change in odds of the PheWAS trait per weighted eGFR-increasing allele. Multiple testing thresholds for significance were set to *p* ≤ 2.75 × 10^−5^(0.05/1813).

### Reporting summary

Further information on research design is available in the Nature Research Reporting Summary linked to this article.

## Supplementary information

Supplementary Information

Supplementary Data 1

Supplementary Data 2

Supplementary Data 3

Supplementary Data 4

Supplementary Data 5

Supplementary Data 6

Supplementary Data 7

Supplementary Data 8

Supplementary Data 9

Description of Additional Supplementary Files

Reporting Summary

## Data Availability

Full summary statistics relating to the Million Veteran Program (MVP) studies are available at dbGAP accession phs001672.v2.p1. Statistically significant reports for S-PrediXcan results for human kidney tissues and PheWAS analyses for eGFR are made available in the supplementary data and tables. Human Kidney RNA-seq data are available at Gene Expression Omnibus (GSE115098).
