# Supplementary material for: Mapping eGFR loci to the renal transcriptome and phenome in the VA Million Veteran Program
Source: Nat Commun. 2019 Aug 26;10:3842. doi: 10.1038/s41467-019-11704-w (PMC6710266; doi:10.1038/s41467-019-11704-w)
Supplement: Supplementary file 1 — Supplementary Information [file 41467_2019_11704_MOESM1_ESM.pdf]

Supplementary Figures

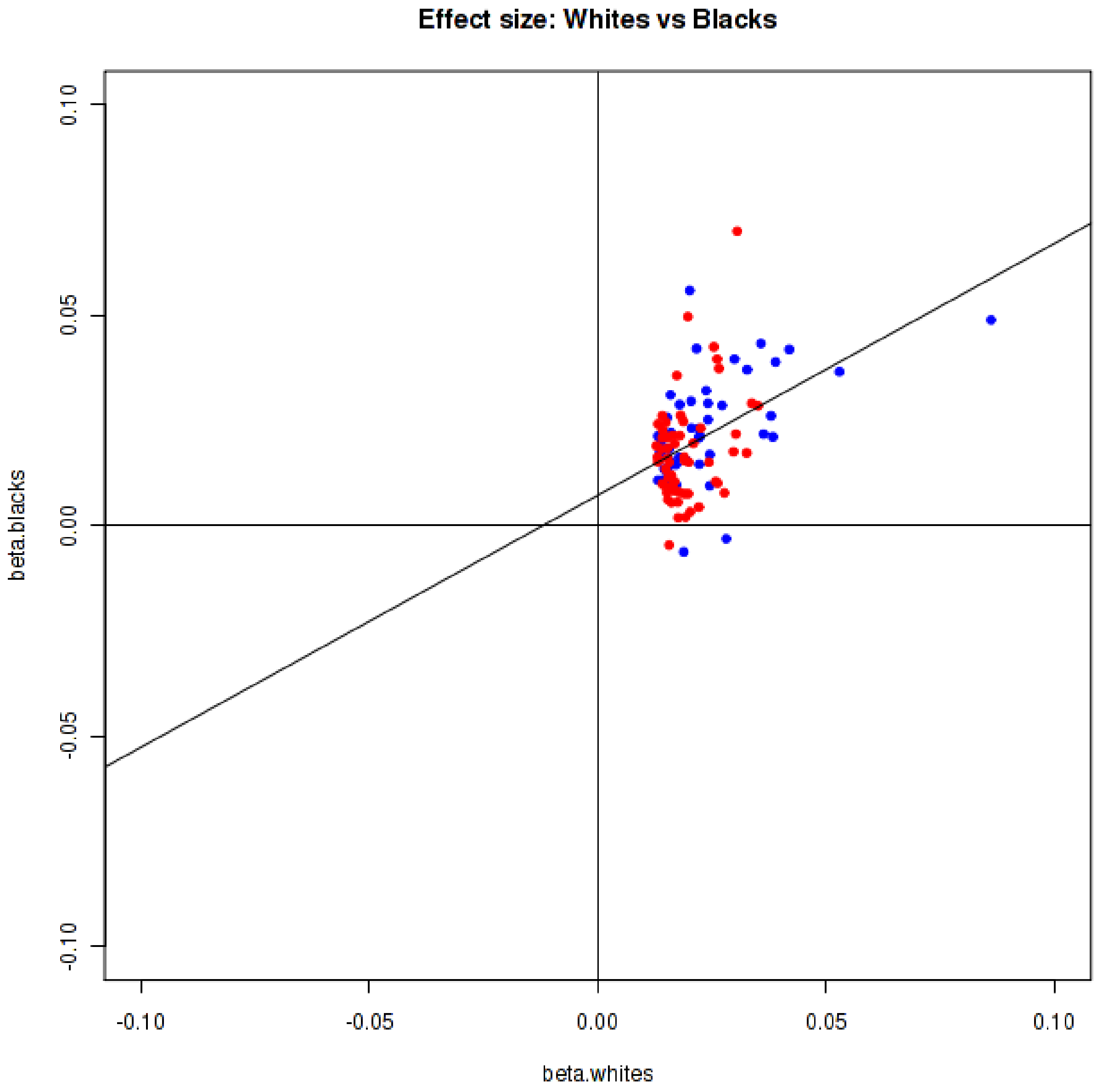

Supplementary Figure 1. Comparison of effect sizes for known and novel SNPs identified with eGFR across non-Hispanic whites and non-Hispanic blacks.

Sentinel SNPs from transethnic discovery meta-analysis were compared for consistency between MVP non-Hispanic blacks (y-axis) and MVP non-Hispanic whites (x-axis). Blue dots denote sentinel SNPs from known loci and red dots denote from novel loci.

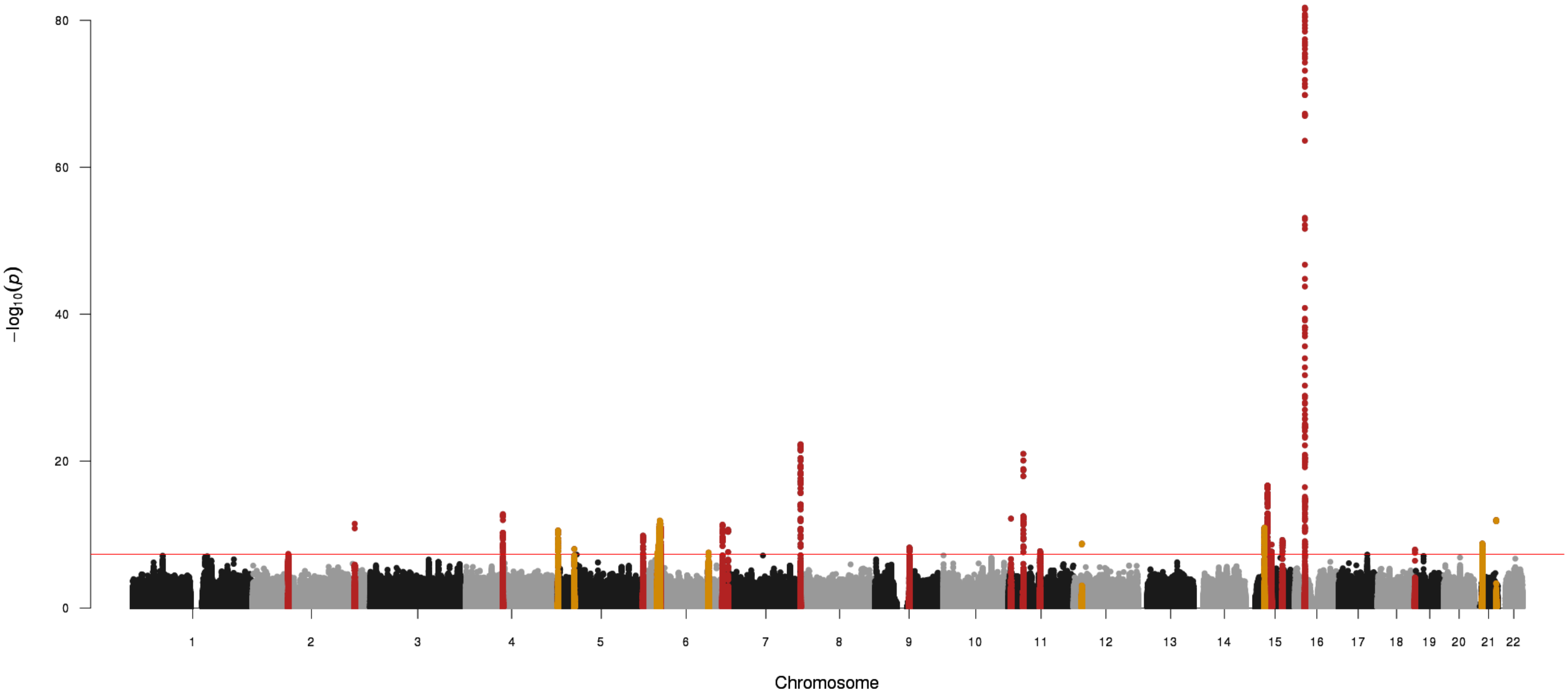

Supplementary Figure 2. Manhattan plot summarizing trans-ethnic discovery meta-analysis of eGFR in diabetic subjects.

The y axis shows the –log10 P-values and the x axis shows the chromosomal positions. The horizontal red line represents the thresholds of P-value = 5 x10-8 for genome-wide significance. SNPs in red are in previously-identified loci, whereas SNPs in orange are in novel loci.

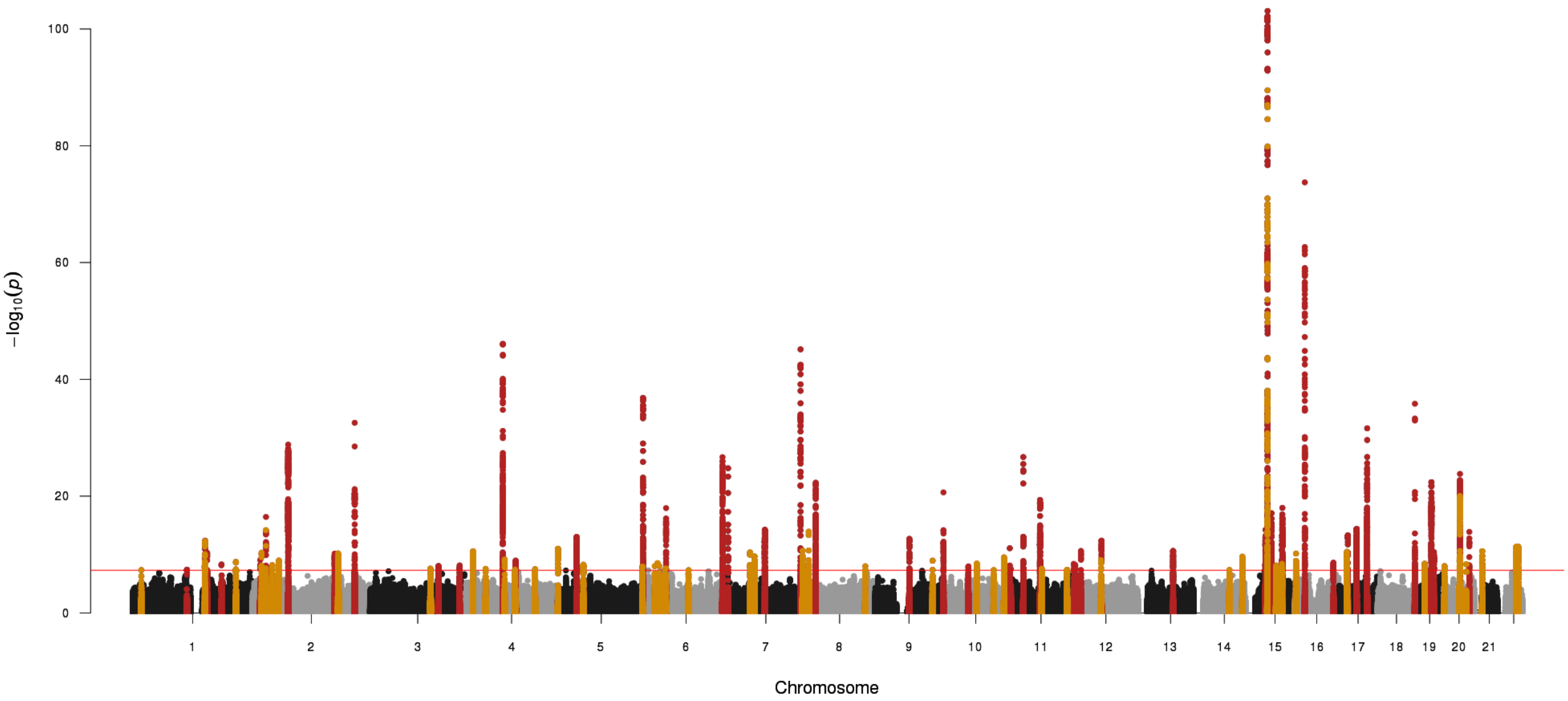

Supplementary Figure 3. Manhattan plot summarizing trans-ethnic discovery meta-analysis of eGFR in non-diabetic subjects.

The y axis shows the –log10 P-values and the x axis shows the chromosomal positions. The horizontal red line represents the thresholds of P-value = 5 x10-8 for genome-wide significance. SNPs in red are in previously-identified loci, whereas SNPs in orange are in novel loci.

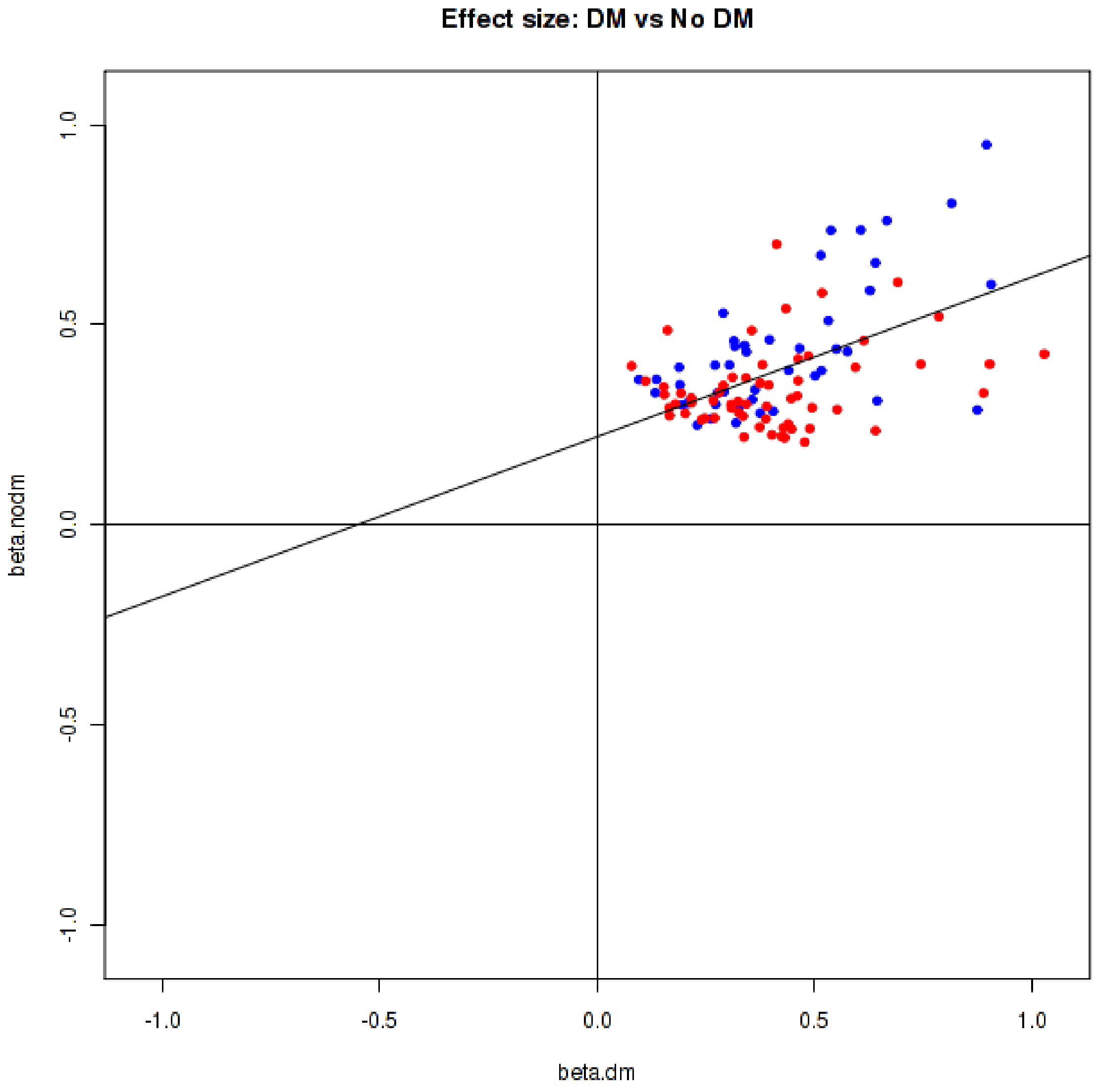

Supplementary Figure 4. Comparison of eGFR SNP effect sizes between diabetic and non-diabetic subjects for known and novel SNPs identified across all subjects.

Sentinel SNPs from transethnic discovery meta-analysis were compared for consistency between MVP diabetic subjects (x-axis) and MVP non-diabetic subjects (y-axis). Blue dots denote sentinel SNPs from known loci and red dots denote from novel loci.

Supplementary Tables

Supplementary Table 1. Conditional association results for all jointly conditional SNPs.

Index SNP						Discovery Results				Conditional Results						
**rsID**	**CHR:BP**	**Nearest Gene**	**Distance**	**Location**	**Effect Allele**	**EAF_{disc}**	**$Effect_{disc}$**	**SE_{disc}**	**$P\text{-value}_{disc}$**	**Lead SNP(s)**	**Novel/Known**	**Nearest Gene(s)**	**R^2**	**$Effect_{cond}$**	**SE_{cond}**	**$P\text{-value}_{cond}$**
rs2048371	2:54870908	SPTBN1		intron	T	0.717	-0.291	0.049	4.24E-09	rs10865282	novel	C2orf73	0.000	-0.276	0.049	2.45E-08
rs1532783	15:76190906	UBE2Q2		intron	T	0.949	-1.066	0.123	4.52E-18	rs11072567	known	NRG4	0.015	-0.783	0.125	3.30E-10
rs199688956	15:76235871	NRG4		untranslated-3	T	0.029	-1.193	0.166	5.72E-13	rs11072567	known	NRG4	0.030	-0.969	0.167	6.09E-09
rs79091515	1:185026908	RNF2		intron	A	0.022	-1.225	0.186	4.65E-11	rs115276619	known	FAM129A	0.443	-1.179	0.186	2.45E-10
rs2102577	4:77287791	CCDC158		intron	A	0.577	0.490	0.046	3.45E-26	rs13146355	known	SHROOM3	0.006	0.453	0.046	1.31E-22
rs35790011	17:19463591	SLC47A1		missense	A	0.058	-1.588	0.250	2.04E-10	rs2252281	known	SLC47A1	*	-1.582	0.250	2.43E-10
rs316020	6:160669081	SLC22A2		intron	A	0.108	0.707	0.072	5.73E-23	rs2279463	known	SLC22A2	0.016	0.670	0.073	3.23E-20
rs11638272	15:39266793	.-.		unknown	C	0.465	0.265	0.045	5.58E-09	rs28833881	novel	.-.	0.003	0.272	0.045	2.18E-09
rs1545715	7:155668309	SHH	63342		A	0.462	-0.264	0.045	3.00E-09	rs288762	novel	SHH	0.002	-0.280	0.045	3.32E-10
rs6976242	7:156125204	LOC285889	105278		A	0.835	-0.346	0.063	3.57E-08	rs288762	novel	SHH	0.000	-0.359	0.063	1.12E-08
rs2762943	20:52790786	CYP24A1	270	near-gene-5	T	0.076	0.495	0.087	1.14E-08	rs35870583	known	BCAS1 -CYP24A1	0.000	0.528	0.087	1.22E-09
rs537808693	6:31396347	MICA	13257		T	0.349	0.342	0.052	5.72E-11	rs532086	known	C2	0.023	0.317	0.053	2.30E-09
rs1136201	17:37879588	ERBB2		missense	A	0.772	0.401	0.054	1.74E-13	rs541524196	known	.-.	0.028	0.332	0.056	2.55E-09
rs34224335	1:150766085	CTSK	2597		A	0.733	-0.409	0.054	4.08E-14	rs543179	novel	MRPS21	0.016	-1.150	0.088	3.90E-39
rs111285796	16:20361087	UMOD		intron	T	0.832	-1.430	0.061	1.42E-121	rs77924615	known	PDILT	0.348	-0.846	0.072	8.29E-32
rs73543348	16:20388957	PDILT		intron	T	0.122	-0.752	0.070	5.23E-27	rs77924615	known	PDILT	0.032	-0.480	0.071	1.19E-11
rs369062552	11:30749169	DCDC5	102756		T	0.963	1.155	0.152	2.97E-14	rs963837	known	MPPED2 -DCDC5	0.055	1.465	0.153	1.24E-21
rs6504021	17:59240473	BCAS3		intron	T	0.764	-0.448	0.058	8.48E-15	rs9895661	known	BCAS3	0.001	-0.348	0.060	6.63E-09
rs11350775	17:59241456	BCAS3		intron	CT	0.122	0.787	0.104	3.84E-14	rs9895661	known	BCAS3	0.001	0.616	0.108	1.21E-08
rs34091020	17:59486437	TBX2		untranslated-3	G	0.493	-0.571	0.056	2.83E-24	rs9895661	known	BCAS3	0.089	-0.418	0.058	5.87E-13

SNPs are ordered by chromosome and position. rsID - dbSNP accession number; CHR:BP - chromosome and build 37 position; CHR:BP - chromosome and build 37 position; Nearest Gene - most proximal gene within 250kb of index SNP; Distance - distance in base pairs from index SNP to nearest gene; Location - location of index SNP relative to nearest gene; Effect allele - allele corresponding to measured effect on the outcome; EAF_{disc} - effect allele frequency in the combined discovery and replication meta-analysis; $Effect_{disc}$ - measured effect in the discovery meta-analysis; SE_{disc} - standard error of the measured effect in the discovery meta-analysis; $P\text{-value}_{disc}$ - association p-value for the measured effect in the discovery meta-analysis; Lead SNP(s) - SNP(s) with most significant association p-value for the measured effect in the discovery meta-analysis on which the SNP in the rsID column was conditioned; Novel/Known - indicator of whether lead SNP locus was previously reported or novel in our analyses; Nearest Gene(s) - most proximal gene within 500kb of Lead SNP(s); R^2 - linkage disequilibrium correlation between SNP in rsID column and Lead SNP(s); $Effect_{cond}$ - measured effect of SNP in the rsID column in the genome-wide joint conditional analysis; SE_{cond} - standard error of the measured effect of SNP in the rsID column in the genome-wide joint conditional analysis; $P\text{-value}_{cond}$ - association p-value for the measured effect of SNP in the rsID column in the genome-wide joint conditional analysis. *rs35790011 monomorphic in 1KG EU

Supplementary Table 2. Association results for all significant eGFR variants from blacks-only MVP analysis.

Index SNP										Discovery Meta-analysis Results			
rsID	CHR:BP	CHR	BP	Nearest Gene	Distance	Location	Effect Allele	Other Allele	Novelty eGFR	$EAF_{Discovery}$	$Effect_{disc}$	SE_{disc}	$P\text{-value}_{comb}$
rs6676150	1:155123837	1	155123837	DPM3	10841		C	G	known	0.303	-0.036	0.006	4.06E-09
rs2279463	6:160668389	6	160668389	SLC22A2			A	G	known	0.807	0.042	0.007	7.50E-10
rs13230509	7:1286192	7	1286192	UNCX	9579		C	G	known	0.283	-0.040	0.006	9.29E-11
rs10265221	7:151414329	7	151414329	PRKAG2			T	C	known	0.824	0.043	0.008	3.78E-08
rs334	11:5248232	11	5248232	HBB			A	T	known	0.057	-0.119	0.014	1.54E-18
rs75113983	11:6156162	11	6156162	OLFR690	16750		A	T	novel	0.976	0.111	0.020	2.25E-08
rs200950799	12:17157119	12	17157119	.-.			T	C	novel	0.011	-1.394	0.232	1.71E-09
rs144803907	15:45158662	15	45158662	C15orf43	90240		A	G	novel	0.119	-0.051	0.009	4.50E-09
rs2486272	15:45672253	15	45672253	GATM			T	C	known	0.225	0.083	0.006	2.02E-39
rs1532783	15:76190906	15	76190906	UBE2Q2			T	C	known	0.904	-0.054	0.009	5.12E-09
rs35790011	17:19463591	17	19463591	SLC47A1			A	G	known	0.058	-0.072	0.012	3.92E-10
rs7212621	17:37527911	17	37527911	FBXL20			T	G	known	0.664	-0.038	0.006	7.51E-11
rs56376587	18:77160235	18	77160235	NFATC1			A	C	known	0.792	0.037	0.007	4.03E-08
rs10084572	21:45412872	21	45412872	AGPAT3	5397		T	C	novel	0.014	-1.378	0.193	1.07E-12

SNPs are ordered by chromosome and position. rsID - dbSNP accession number; CHR:BP - chromosome and build 37 position; Nearest Gene - most proximal gene within 250kb of index SNP; Distance - distance in base pairs from index SNP to nearest gene; Location - location of index SNP relative to nearest gene; Effect allele - allele corresponding to measured effect on the outcome; Other allele - allele not corresponding to measured effect on the outcome; Novelty eGFR – annotation of whether locus is novel or previously identified in a GWAS of eGFR; EAF_{disc} - effect allele frequency in the combined discovery and replication meta-analysis; $Effect_{disc}$ - measured effect in the discovery meta-analysis; SE_{disc} - standard error of the measured effect in the discovery meta-analysis; $P\text{-value}_{disc}$ - association p-value for the measured effect in the discovery meta-analysis.

Supplementary Table 3. Summary statistics for regression of effect estimates between MVP race/ethnic groups at known and novel SNPs.

Variable	SNP Group	slope	r2
	All	0.598	0.212
	Novel	0.786	0.132
**Effect size**	Known	0.485	0.254
	All	0.988	0.611
	Novel	1.011	0.666
**Frequency**	Known	0.943	0.518
	All	0.186	0.121
	Novel	-0.827	0.153
**Variance**	Known	0.14	0.104

Variable – parameter being compared across races; SNP group – all SNPs, novel only, or known only; slope – slope of the best-fit regression line for comparison of parameters between non-Hispanic whites and non-Hispanic Blacks; r2 - denotes correlation between effect estimates calculated from a linear regression model.

Supplementary Table 4. LD Score Regression results for each contributing analysis set.

Race Group	Phenotype group	N Samples	Lambda	LD Intercept (SE)	H2 (SE)	N SNPs
Whites	DM+ HTN+	64,389	1.1113	1.0242 (0.0078)	0.0939 (0.0104)	1164108
	DM- HTN+	89,838	1.1619	1.04 (0.0087)	0.1118 (0.0103)	1164794
	DM- HTN-	56,146	1.1459	1.028 (0.0096)	0.1523 (0.0172)	1169086
	DM+ HTN-	6,212	1.0105	0.9909 (0.0062)	0.1279 (0.0687)	1174082
Blacks	DM+ HTN+	19,428	1.0255	1.0086 (0.0033)	0.0556 (0.0204)	11565520
	DM- HTN+	23,066	1.0315	1.0189 (0.0039)	0.0546 (0.0245)	11560578
	DM- HTN-	12,265	1.0165	0.999 (0.0034)	0.1249 (0.0365)	11572037
	DM+ HTN-	1,494	0.9927	0.9975 (0.0034)	Not calculated	11588764

Results presented by Race Group (Non-Hispanic whites, non-Hispanic blacks) and phenotype group (diabetes y/n and hypertension y/n). Lambda – inflation factor of GWAS in each strata: median(chi^2)/0.4549; LD intercept (SE) – intercept from LD score regression analysis with standard error; H2 (SE) – heritability and standard error as computed from LD score regression; N SNPs – number of SNPs included in analysis after merging with race-specific LD reference data.

Supplementary Table 5. **Expression from single-cell RNA sequencing of murine kidney cell types of mouse homologs of colocalized genes associated with eGFR.**

Gene	Endo	Podo	PT	LOH	DCT	CD-PC	CD-IC	Fib	Macro	Neutro	B lymph	T lymph	NK
Bst2	1.395	-0.625	-0.648	-0.536	-0.544	-0.562	-0.455	2.736	1.488	-0.580	0.245	-0.214	-0.366
Nars2	-0.364	2.611	0.337	0.467	0.774	0.249	0.087	-1.141	-0.162	0.268	0.077	0.050	-0.486
Arnt	0.618	2.096	0.271	0.545	0.805	0.589	0.085	-0.939	0.041	-0.120	0.477	-1.020	-0.894
Shroom3	-0.487	1.512	0.087	0.715	0.719	1.305	0.354	-1.059	-0.410	0.083	-0.510	-0.986	-0.891
Mettl10	0.570	1.441	0.177	1.586	1.287	0.517	0.989	-1.572	-0.896	-0.157	-1.028	-1.318	-0.829
Tprkb	0.278	-0.308	2.080	-0.341	-0.252	-0.507	-0.534	-0.765	0.036	0.583	-0.660	-0.647	-0.474
Arl16	0.496	0.131	0.810	0.826	1.419	0.932	0.375	-1.145	-0.108	0.778	-0.590	-0.741	-1.061
Angptl3	-0.293	0.063	0.964	0.935	1.257	0.452	0.511	-1.138	-0.069	1.034	-0.243	-0.917	-0.716
Spire2	-0.375	0.044	0.472	0.601	1.109	1.075	0.920	-1.186	-0.075	0.747	-0.311	-0.890	-0.785
Ube2q2	-0.001	-0.210	0.588	0.750	0.852	0.844	0.651	-1.184	-0.194	0.290	-0.623	-0.491	0.043
Klhdc7a	-0.481	-0.475	0.188	0.555	0.714	1.682	0.529	-0.877	-0.157	0.259	-0.626	-0.877	-0.818
Nrip1	-0.277	-0.442	0.043	0.161	-0.028	1.388	-0.199	-1.249	0.927	-1.107	-1.576	0.448	0.643
Usp24	-0.444	-0.403	0.681	0.950	1.008	1.359	0.033	-1.816	-1.014	0.797	-0.025	-0.783	-0.942
Manba	-0.595	0.260	0.812	0.478	0.868	1.223	0.165	-1.201	0.490	-0.143	-0.698	-0.636	-1.118
Rnf152	-0.423	-0.437	-0.068	-0.307	0.082	-0.279	2.094	-0.586	-0.334	-0.356	-0.381	-0.640	-0.635
Gbas	-0.241	0.047	-0.526	1.143	0.820	1.305	1.440	-0.896	-0.553	-0.079	-1.010	-0.790	-0.738
Whamm	-0.157	0.457	0.308	0.649	1.012	0.198	0.332	-1.338	1.393	0.033	0.002	-0.103	-0.602
Rgs14	-0.967	-0.409	0.242	0.426	1.123	0.082	-0.214	-0.662	0.277	1.966	-0.017	0.079	1.594
Rnaseh2c	-0.376	-0.663	-0.350	-0.622	-1.049	-0.808	-0.963	0.152	0.093	-0.010	0.049	0.877	0.887
Sf3b2	0.488	-0.490	-0.913	-0.632	-0.816	-0.364	-0.359	0.183	-0.075	-0.872	0.353	0.556	0.478

Gene - mouse homolog of significant gene identified in kidney tissue (see ST7a). Endo - endothelial; Podo - podocyte; PT - proximal tubule; LOH - Loop of Henle; DCT - distal convoluted tubule; CD-PC - collecting duct principal cell; CD-IC - collecting duct intercalated cell; Fib - fibroblast; Macro - macrophage; Neutro - neutrophil; B lymph - B lymphocyte; T lymph - T lymphocyte; NK - natural killer cell.

Supplementary Table 6. **Expression of genes from mouse scRNA-seq in human kidney from the Human Protein Atlas.**

Gene	Glomeruli expression[a]	Tubule Expression[a]
BST2	High	Medium
NARS2	Medium	High
ARNT	Medium	Low
SHROOM3	Low	Medium
METTL10	Medium	Medium
TPRKB	Low	Medium
ARL16	Not detected	High
ANTPTL3	Not detected	Not detected
SPIRE2	High	Medium
UBE2Q2	NA[b]	NA[b]
KLHDC7A	NA[c]	NA[c]
NRIP1	Medium	Medium
USP24	Not detected	Medium
MANBA	Not detected	Medium
RNF152	NA[b]	NA[b]
GBAS	Medium	High
WHAMM	Medium	Medium
RGS14	Not detected	Medium
RNASEH2C	NA[b]	NA[b]
SF3B2	Medium	High

Expression in glomeruli and tubule cells categorized as high, medium, or low. [a]Expression profiles for proteins in human kidney based on immunohistochemisty using tissue micro arrays. [b]Pending normal tissue annotation. [c]Estimation of protein expression could not be performed. View primary data.

Supplementary Table 7: 63 SNPs included in w-GRS construction using only previously-published CKDgen results.

SNP	Chr	Position (bp)	Index Gene	Effect allele	Other Allele	Effect	SE	P-value	Index
rs7546668	1	15855123	CASP9	C	G	-0.0063	0.001	1.14E-09	Gorski
rs10127790	1	109891133	SYPL2	T	C	0.0061	0.001	7.58E-09	Gorski
rs267738	1	150940625	ANXA9	T	G	-0.0091	0.0011	1.48E-14	Gorski
rs3850625	1	201016296	CACNA1S	A	G	0.0078	0.0013	5.53E-10	Pattaro
rs807601	2	15793014	DDX1	T	G	0.0064	0.0009	6.60E-12	Pattaro
rs780093	2	27742603	GCKR	T	C	0.0081	0.0009	1.57E-16	Gorski
rs6546838	2	73679280	ALMS1	A	G	-0.0093	0.001	7.72E-20	Pattaro
rs7422339 (now rs1047891)	2	211540507	CPS1	A	C	-0.0106	0.001	2.18E-23	Pattaro
rs2861422	3	141724644	TFDP2	T	C	0.0074	0.001	9.12E-14	Pattaro
rs10513801	3	185822353	ETV5	T	G	0.007	0.0012	2.47E-09	Pattaro
rs17319721	4	77368847	SHROOM3	A	G	-0.0114	0.0009	1.32E-37	Pattaro
rs11959928	5	39397132	DAB2	A	T	-0.0083	0.0009	1.66E-20	Pattaro
rs6420094	5	176817636	SLC34A1	A	G	0.0096	0.001	4.92E-22	Pattaro
rs9472135	6	43809802	VEGFA	T	C	-0.008	0.001	3.34E-15	Pattaro
rs316009	6	160675764	SLC22A2	T	C	0.0131	0.0014	4.38E-19	Pattaro
rs10277115	7	1285195	UNCX	A	T	0.009	0.0012	8.72E-14	Pattaro
rs848490	7	77555005	TMEM60	C	G	0.0073	0.001	7.80E-13	Pattaro
rs7805747	7	151407801	PRKAG2	A	G	-0.013	0.0011	7.96E-29	Pattaro
rs36071802	8	23715871	STC1	T	C	0.0079	0.0009	1.16E-15	Gorski
rs10746942	9	71434465	PIP5K1B	A	G	0.0086	0.0009	3.56E-18	Gorski
rs80282103	10	899071	WDR37	A	T	0.0123	0.0017	1.12E-11	Gorski
rs10994860	10	52645424	A1CF	T	C	0.0071	0.001	1.66E-12	Pattaro
rs163160	11	2789955	KCNQ1	A	G	0.0064	0.001	1.72E-10	Pattaro
rs963837	11	30749090	MPPED2	T	C	-0.0078	0.0009	5.69E-18	Pattaro
rs4014195	11	65506822	AP5B1	C	G	0.0055	0.0008	1.10E-11	Pattaro
rs10774021	12	349298	SLC6A13	T	C	-0.0063	0.0009	4.77E-12	Pattaro
rs10491967	12	3368093	TSPAN9	A	G	-0.0095	0.0013	5.18E-14	Pattaro
rs9529913	13	72345089	DACH1	T	C	-0.0066	0.0009	2.51E-11	Gorski
rs2453533	15	45641225	GATM	A	C	-0.0135	0.0009	2.65E-43	Gorski
rs491567	15	53946593	WDR72	A	C	-0.0084	0.001	2.86E-15	Pattaro
rs1394125	15	76158983	UBE2Q2	A	G	-0.0073	0.001	5.47E-14	Pattaro
rs13329952	16	20366507	UMOD	T	C	-0.0158	0.0011	9.47E-43	Pattaro
rs894680	17	19440538	SLC47A1	A	G	-0.0074	0.001	5.46E-12	Gorski
rs12451586	17	37633835	CDK12	A	T	-0.0092	0.0011	2.78E-15	Gorski
rs11657044	17	59450105	BCAS3	T	C	-0.0115	0.0012	7.89E-22	Pattaro
rs71359461	18	77156103	NFATC1	C	G	-0.0086	0.0013	3.67E-10	Gorski
rs12460876	19	33356891	SLC7A9	T	C	-0.0066	0.0009	1.86E-13	Pattaro
rs6058093	20	33213196	TP53INP2	A	C	-0.0074	0.001	2.26E-13	Gorski
rs6127099	20	52731402	BCAS1	A	T	-0.0095	0.0011	2.91E-17	Gorski
rs10874312	1	82944571	LPHN2	A	G	-0.0057	0.0011	2.20E-08	Gorski
rs12144044	1	113248791	RHOC	A	C	-0.0061	0.0011	2.87E-08	Gorski

rs187355703	2	176993583	HOXD8	C	G	0.0182	0.003	5.15E-10	Gorski
rs111366116	5	53295546	ARL15	T	C	0.0094	0.0015	6.27E-10	Gorski
rs113246091	5	67739274	PIK3R1	A	G	-0.0095	0.0016	1.98E-09	Gorski
rs7764488	6	133812872	EYA4	A	G	0.0061	0.0011	4.08E-09	Gorski
rs13298297	9	119264108	ASTN2	A	G	-0.0075	0.0014	1.53E-08	Gorski
rs1111571	16	68363181	SLC7A6	A	G	0.0061	0.0011	6.20E-09	Gorski
rs9962915	18	5593171	EPB41L3	T	C	-0.0055	0.001	7.19E-09	Gorski
rs12458009	18	59350507	RNF152	T	G	-0.0064	0.0012	2.90E-08	Gorski
rs2802729	1	243501763	SDCCAG8	A	C	-0.0046	0.0008	2.20E-08	Pattaro
rs2712184	2	217682779	IGFBP5	A	C	-0.0048	0.0008	3.02E-09	Pattaro
rs6795744	3	13906850	WNT7A	A	G	0.006	0.0011	3.33E-08	Pattaro
rs228611	4	103561709	NFKB1	A	G	-0.0056	0.0008	3.58E-12	Pattaro
rs3750082	7	32919927	KBTBD2	A	T	0.0045	0.0008	3.22E-08	Pattaro
rs6459680	7	156258568	RNF32	T	G	-0.0055	0.0009	1.07E-09	Pattaro
rs1106766	12	57809456	INHBC	T	C	0.0061	0.001	2.41E-09	Pattaro
rs476633	15	41392134	INO80	C	G	0.0051	0.0009	8.90E-09	Pattaro
rs164748	16	89708292	DPEP1	C	G	0.0046	0.0008	1.95E-08	Pattaro NDM only
rs11666497	19	38464262	SIPA1L3	T	C	-0.0058	0.0011	4.25E-08	Pattaro
rs4667594	2	170008506	LRP2	A	T	-0.0044	0.0008	3.52E-08	Pattaro
rs9682041	3	170091902	SKIL	T	C	-0.0068	0.0012	2.58E-08	Pattaro NDM only
rs7759001	6	27341409	ZNF204	A	G	-0.0051	0.0009	1.75E-08	Pattaro
rs7956634	12	15321194	PTPRO	T	C	-0.0068	0.001	7.17E-12	Pattaro

SNP – dbSNP accession number; Chr – chromosome; Position (bp) – build 37 position of each SNP; Index Gene – Gene annotation from publication; Effect allele - - allele corresponding to measured effect on the outcome; Other allele - allele not corresponding to measured effect on the outcome; Effect - measured effect in the published paper; SE - standard error of the measured effect in the published paper; P-value - association p-value for the measured effect in the published paper; Index – Manuscript reporting associations for which summary statistics are derived, Gorski = Gorski M et al, Scientific Reports, 2017 Apr 28, PMID 28452372 [1]; Pattaro = Pattaro C et al, Nature Communications, 2016 Jan 21, PMID 26831199[2]

Supplementary Table 8. Suggestive phenome-wide associations of previously published CKDgen-weighted eGFR genetic risk score (GRS) in unrelated white individuals in MVP.

PheCode	Description	Phenotype Group	N_{Total}	N_{Cases}	$N_{Controls}$	OR	SE	P-value
585.3	Chronic renal failure [CKD]	genitourinary	167997	16305	151692	0.876	0.008	**3.55E-57**
585	Renal failure	genitourinary	173079	21387	151692	0.893	0.007	**6.32E-53**
585.33	Chronic Kidney Disease, Stage III	genitourinary	158973	7281	151692	0.885	0.012	**6.13E-24**
401.22	Hypertensive chronic kidney disease	circulatory system	55004	8490	46514	0.917	0.012	**5.19E-13**
594	Urinary calculus	genitourinary	186563	13178	173385	1.062	0.009	**2.75E-11**
594.1	Calculus of kidney	genitourinary	184433	11048	173385	1.064	0.010	**3.12E-10**
401.2	Hypertensive heart and/or renal disease	circulatory system	56739	10225	46514	0.939	0.011	**1.72E-08**
586	Other disorders of the kidney and ureters	genitourinary	160917	9225	151692	0.949	0.011	**1.11E-06**
594.3	Calculus of ureter	genitourinary	176071	2686	173385	1.088	0.020	**1.59E-05**

Table is sorted by p-value. PheCode - PheWAS code, a hierarchical grouping of International Classification of Disease, 9th edition (ICD9) codes applied to EMR data, which loosely follow the 3-digit (category) and section groupings defined with the ICD9 code system itself, and have been revised based on statistical co-occurrence, code frequency, and human review; Description - full name of PheCode grouping; Phenotype Group - physiological system to which the PheCode is assigned; N_{Total} - total number of individuals not excluded in analysis of PheCode; N_{Cases} -number of individuals with one or more diagnosis codes corresponding to the PheCode; $N_{controls}$ -number of individuals lacking diagnosis codes or exclusion criteria corresponding to the PheCode; OR -measured odds ratio per standard deviation of w-GRS (standard deviation for all three analyses = 0.0377) for association between the weighted GRS and PheCode; SE - standard error of the measured effect; P-value - p-value for association of the weighted GRS and the PheCode.

Supplementary Note 1.

Relationship between t-statistic, chi-square and R^2

$$t = r \times \sqrt{\frac{n-2}{1-r^2}} \qquad (1)$$

Rearranging the equation in terms of r^2

$$r^2 = \frac{t^2}{(n-2)+t^2} \qquad (2)$$

When n is large enough (n > 20) t-distribution approximates the z distribution

$$r^2 \approx \frac{z^2}{(n-2)+z^2} \qquad (3)$$

The square of a z distribution is the Chi-square distribution

$$r^2 \approx \frac{\chi^2}{(n-2)+\chi^2} \qquad (4)$$

Supplementary Equation 1 describes the relationship between a student's t statistic, correlation coefficient r, and r^2. Supplementary Equation 4 describes the transformed equation that describes r^2 in terms of the chi-square.

As n → ∞

$$r^2 \approx \frac{\chi^2}{n} \qquad (5)$$

When the sample size is sufficiently large enough, the variance explained by each SNP can then be well approximated by Supplementary Equation 5.

$$R^2 \approx \sum_{i=1}^{m} \frac{{\chi_i}^2}{n_i} \qquad (6)$$

Where m = total number of independent SNPs in the study, R^2 is the total variance explained by independent SNPs, n_i and ${\chi_i}^2$ represent the number individuals in the analysis and the square of the Wald z-statistic for the given SNP, respectively.

Supplementary Methods

Enrichment analyses in DEPICT[3] were conducted using significant GWAS sentinel SNPs from three separate analyses as input: 1) transethnic analyses of all MVP subjects, 2) transethnic analyses of MVP subjects with DM, and 3) transethnic analyses of MVP subjects without DM. DEPICT is based on predefined phenotypic gene sets from multiple databases and Affymetrix

HGU133a2.0 expression microarray data from more than >37k subjects to build highly-expressed gene sets for Medical Subject Heading (MeSH) tissue and cell type annotations. Output includes a p-value for enrichment and a yes/no indicator of whether the FDR q-value is <0.05. Tissue level and gene-set enrichment features are considered.

Supplementary Discussion

We conducted tissue-specific and pathway gene enrichment analyses using DEPICT software[3] using the significant GWAS sentinel SNPs identified from transethnic analyses of all subjects, diabetics participants, and non-diabetic participants, respectively, (Supplementary Data 6–7 and 8–9). Enrichment analyses of GWAS SNPs from transethnic two-stage analyses of diabetic participants failed due to an insufficient number of independent loci annotated by DEPICT gene sets. No significant (FDR < 5%) tissue-specific or pathway gene set enrichment was detected. The two most significant tissue-specific gene set enrichments were observed in the urinary tract and the kidney in all subjects ($p = 4.96x10^{-3}$ and $p = 6.49x10^{-3}$, respectively) and non-diabetics participants ($p = 8.90x10^{-4}$ and $1.14x10^{-3}$, respectively). The most significant pathway gene set enrichment was observed in abnormal placental labyrinth vasculature morphology for all subjects (MP:0008803; $p = 3.02x10^{-6}$) and in abnormal liver morphology for non-diabetic participants (MP:0000598; $p = 1.51x10^{-5}$).

Supplementary Note 2.

<u>Million Veteran Program: Consortium Acknowledgement</u>

<u>MVP Executive Committee</u>

- Co-Chair: J. Michael Gaziano, M.D., M.P.H.
- Co-Chair: Rachel Ramoni, D.M.D., Sc.D.
- Jim Breeling, M.D. (ex-officio)
- Kyong-Mi Chang, M.D.
- Grant Huang, Ph.D.
- Sumitra Muralidhar, Ph.D.
- Christopher J. O'Donnell, M.D., M.P.H.
- Philip S. Tsao, Ph.D.

<u>MVP Program Office</u>

- Sumitra Muralidhar, Ph.D.
- Jennifer Moser, Ph.D.

<u>MVP Recruitment/Enrollment</u>

- Recruitment/Enrollment Director/Deputy Director, Boston – Stacey B. Whitbourne, Ph.D.; Jessica V. Brewer, M.P.H.
- MVP Coordinating Centers
    - Clinical Epidemiology Research Center (CERC), West Haven – John Concato, M.D., M.P.H.

    - Cooperative Studies Program Clinical Research Pharmacy Coordinating Center, Albuquerque - Stuart Warren, J.D., Pharm D.; Dean P. Argyres, M.S.
    - Genomics Coordinating Center, Palo Alto – Philip S. Tsao, Ph.D.
    - Massachusetts Veterans Epidemiology Research Information Center (MAVERIC), Boston - J. Michael Gaziano, M.D., M.P.H.
    - MVP Information Center, Canandaigua – Brady Stephens, M.S.
- Core Biorepository, Boston – Mary T. Brophy M.D., M.P.H.; Donald E. Humphries, Ph.D.
- MVP Informatics, Boston – Nhan Do, M.D.; Shahpoor Shayan
- Data Operations/Analytics, Boston – Xuan-Mai T. Nguyen, Ph.D.

MVP Science

- Genomics - Christopher J. O'Donnell, M.D., M.P.H.; Saiju Pyarajan Ph.D.; Philip S. Tsao, Ph.D.
- Phenomics - Kelly Cho, M.P.H, Ph.D.
- Data and Computational Sciences – Saiju Pyarajan, Ph.D.
- Statistical Genetics – Elizabeth Hauser, Ph.D.; Yan Sun, Ph.D.; Hongyu Zhao, Ph.D.

MVP Local Site Investigators

- Atlanta VA Medical Center (Peter Wilson)
- Bay Pines VA Healthcare System (Rachel McArdle)
- Birmingham VA Medical Center (Louis Dellitalia)
- Cincinnati VA Medical Center (John Harley)
- Clement J. Zablocki VA Medical Center (Jeffrey Whittle)
- Durham VA Medical Center (Jean Beckham)
- Edith Nourse Rogers Memorial Veterans Hospital (John Wells)
- Edward Hines, Jr. VA Medical Center (Salvador Gutierrez)
- Fayetteville VA Medical Center (Gretchen Gibson)
- VA Health Care Upstate New York (Laurence Kaminsky)
- New Mexico VA Health Care System (Gerardo Villareal)
- VA Boston Healthcare System (Scott Kinlay)
- VA Western New York Healthcare System (Junzhe Xu)
- Ralph H. Johnson VA Medical Center (Mark Hamner)
- Wm. Jennings Bryan Dorn VA Medical Center (Kathlyn Sue Haddock)
- VA North Texas Health Care System (Sujata Bhushan)
- Hampton VA Medical Center (Pran Iruvanti)
- Hunter Holmes McGuire VA Medical Center (Michael Godschalk)
- Iowa City VA Health Care System (Zuhair Ballas)
- Jack C. Montgomery VA Medical Center (Malcolm Buford)
- James A. Haley Veterans' Hospital (Stephen Mastorides)
- Louisville VA Medical Center (Jon Klein)
- Manchester VA Medical Center (Nora Ratcliffe)
- Miami VA Health Care System (Hermes Florez)
- Michael E. DeBakey VA Medical Center (Alan Swann)

- Minneapolis VA Health Care System (Maureen Murdoch)
- N. FL/S. GA Veterans Health System (Peruvemba Sriram)
- Northport VA Medical Center (Shing Shing Yeh)
- Overton Brooks VA Medical Center (Ronald Washburn)
- Philadelphia VA Medical Center (Darshana Jhala)
- Phoenix VA Health Care System (Samuel Aguayo)
- Portland VA Medical Center (David Cohen)
- Providence VA Medical Center (Satish Sharma)
- Richard Roudebush VA Medical Center (John Callaghan)
- Salem VA Medical Center (Kris Ann Oursler)
- San Francisco VA Health Care System (Mary Whooley)
- South Texas Veterans Health Care System (Sunil Ahuja)
- Southeast Louisiana Veterans Health Care System (Amparo Gutierrez)
- Southern Arizona VA Health Care System (Ronald Schifman)
- Sioux Falls VA Health Care System (Jennifer Greco)
- St. Louis VA Health Care System (Michael Rauchman)
- Syracuse VA Medical Center (Richard Servatius)
- VA Eastern Kansas Health Care System (Mary Oehlert)
- VA Greater Los Angeles Health Care System (Agnes Wallbom)
- VA Loma Linda Healthcare System (Ronald Fernando)
- VA Long Beach Healthcare System (Timothy Morgan)
- VA Maine Healthcare System (Todd Stapley)
- VA New York Harbor Healthcare System (Scott Sherman)
- VA Pacific Islands Health Care System (Gwenevere Anderson)
- VA Palo Alto Health Care System (Philip Tsao)
- VA Pittsburgh Health Care System (Elif Sonel)
- VA Puget Sound Health Care System (Edward Boyko)
- VA Salt Lake City Health Care System (Laurence Meyer)
- VA San Diego Healthcare System (Samir Gupta)
- VA Southern Nevada Healthcare System (Joseph Fayad)
- VA Tennessee Valley Healthcare System (Adriana Hung)
- Washington DC VA Medical Center (Jack Lichy)
- W.G. (Bill) Hefner VA Medical Center (Robin Hurley)
- White River Junction VA Medical Center (Brooks Robey)
- William S. Middleton Memorial Veterans Hospital (Robert Striker)

Supplementary Note 3.

Members of the CKDGen Consortium and collaborators contributing to the eGFR GWAS meta-analysis and follow-up

Matthias Wuttke[1,2], Yong Li[1], Man Li[3], Karsten B. Sieber[4], Mary F. Feitosa[5], Mathias Gorski[6,7], Adrienne Tin[8,9], Lihua Wang[5], Audrey Y. Chu[10], Anselm Hoppmann[1], Holger Kirsten[11,12], Ayush Giri[13,14], Jin-Fang Chai[15], Gardar Sveinbjornsson[16], Bamidele O. Tayo[17], Teresa Nutile[18],

Christian Fuchsberger[19], Jonathan Marten[20], Massimiliano Cocca[21], Sahar Ghasemi[22,23], Yizhe Xu[3], Katrin Horn[11,12], Damia Noce[19], Peter J. van der Most[24], Sanaz Sedaghat[25], Zhi Yu[8,26], Masato Akiyama[27, 28], Saima Afaq[29,30], Tarunveer S. Ahluwalia[31], Peter Almgren[32], Najaf Amin[25], Johan Ärnlöv[33,34], Stephan J. L. Bakker[35], Nisha Bansal[36,37], Daniela Baptista[38], Sven Bergmann[39,40,41], Mary L. Biggs[42,43], Ginevra Biino[44], Michael Boehnke[45], Eric Boerwinkle[46], Mathilde Boissel[47], Erwin P. Bottinger[48,49], Thibaud S. Boutin[20], Hermann Brenner[50,51], Marco Brumat[52], Ralph Burkhardt[12,53,54], Adam S. Butterworth[55,56], Eric Campana[52], Archie Campbell[57], Harry Campbell[58], Mickaël Canouil[47], Robert J. Carroll[59], Eulalia Catamo[21], John C. Chambers[29,60,61,62,63], Miao-Ling Chee[64,] Miao-Li Chee[64], Xu Chen[65], Ching-Yu Cheng[64,66,67], Yurong Cheng[1], Kaare Christensen[68], Renata Cifkova[69,70], Marina Ciullo[18,71], Maria Pina Concas[21], James P. Cook[72], Josef Coresh[8], Tanguy Corre[39,40,73], Cinzia Felicita Sala[74], Daniele Cusi[75,76], John Danesh[77], E. Warwick Daw[5], Martin H. de Borst[35], Alessandro De Grandi[19], Renée de Mutsert[78], Aiko P. J. de Vries[79], Frauke Degenhardt[80], Graciela Delgado[81], Ayse Demirkan[25], Emanuele Di Angelantonio[82,83], Katalin Dittrich[84,85], Jasmin Divers[86], Rajkumar Dorajoo[87], Kai-Uwe Eckardt[88,89], Georg Ehret[38], Paul Elliott [90,91,92,93], Karlhans Endlich[23,94], Michele K. Evans[95], Janine F. Felix[25,96,97], Valencia Hui Xian Foo[64], Oscar H. Franco[25,98], Andre Franke[80], Barry I. Freedman[99], Sandra Freitag-Wolf[100], Yechiel Friedlander[101], Philippe Froguel[47,102], Ron T. Gansevoort[35], He Gao[90], Paolo Gasparini[21,52], J. Michael Gaziano[103], Vilmantas Giedraitis[104], Christian Gieger[105,106,107], Giorgia Girotto[21,52], Franco Giulianini[108], Martin Gögele[19], Scott D. Gordon[109], Daniel F. Gudbjartsson[16], Vilmundur Gudnason[110,111], Toomas Haller[112], Pavel Hamet[113,114], Tamara B. Harris[115], Catharina A. Hartman[116], Caroline Hayward[20], Jacklyn N. Hellwege[117,118,119,] Chew-Kiat Heng[120,121], Andrew A. Hicks[19], Edith Hofer[122,123], Wei Huang[124,125], Nina Hutri-Kähönen[126,127], Shih-Jen Hwang[128,129], M. Arfan Ikram[25], Olafur S. Indridason[130], Erik Ingelsson[131,132,133,134], Marcus Ising[135], Vincent W. V. Jaddoe [25,96,97], Johanna Jakobsdottir[136], Jost B. Jonas [137,138], Peter K. Joshi [58], Navya Shilpa Josyula [139], Bettina Jung[6], Mika Kähönen[140,141], Yoichiro Kamatani[27,142], Candace M. Kammerer[143], Masahiro Kanai[27,144], Mika Kastarinen[145], Shona M. Kerr[20], Chiea-Chuen Khor[64,87], Wieland Kiess[12,84,85], Marcus E. Kleber [81], Wolfgang Koenig[146,147,148], Jaspal S. Kooner[61,62,63,149], Antje Körner[12,84,85], Peter Kovacs[150], Aldi T. Kraja[5], Alena Krajcoviechova[69,70], Holly Kramer[17,151], Bernhard K. Krämer[81], Florian Kronenberg[152], Michiaki Kubo[153], Brigitte Kühnel[105], Mikko Kuokkanen[154,155], Johanna Kuusisto[145,156], Martina La Bianca[21], Markku Laakso[145,156], Leslie A. Lange[157], Carl D. Langefeld[86], Jeannette Jen-Mai Lee[15], Benjamin Lehne[29], Terho Lehtimäki[158,159], Wolfgang Lieb[160], Lifelines Cohort Study[161], Su-Chi Lim[15,162], Lars Lind[163], Cecilia M. Lindgren[164,165], Jun Liu[25], Jianjun Liu[87,166], Markus Loeffler[11,12], Ruth J. F. Loos[48,167], Susanne Lucae[135], Mary Ann Lukas[168], Leo-Pekka Lyytikäinen[158,159], Reedik Mägi[112], Patrik K. E. Magnusson [65], Anubha Mahajan[169,170], Nicholas G. Martin[109], Jade Martins[171], Winfried März[172,173,174], Deborah Mascalzoni[19], Koichi Matsuda[175], Christa Meisinger[176,177], Thomas Meitinger[147,178,179], Olle Melander[180], Andres Metspalu[112], Evgenia K. Mikaelsdottir[16], Yuri Milaneschi[181,] Kozeta Miliku[25,96,97], Pashupati P. Mishra[158,159], V. A. Million Veteran Program[161], Karen L. Mohlke[182], Nina Mononen[158,159], Grant W. Montgomery[183], Dennis O. Mook-Kanamori[78,184], Josyf C. Mychaleckyj[185], Girish N. Nadkarni[48,186], Mike A. Nalls[187,188], Matthias Nauck[23,189], Kjell Nikus[190,191], Boting Ning[192], Ilja M. Nolte[24], Raymond Noordam[193], Jeffrey O'Connell[194], Michelle L. O'Donoghue[195,196], Isleifur Olafsson[197], Albertine J. Oldehinkel[116], Marju Orho-Melander[32], Willem H. Ouwehand[77], Sandosh Padmanabhan[198], Nicholette D. Palmer[199], Runolfur Palsson[111,130], Brenda W. J. H. Penninx[181], Thomas Perls[200], Markus Perola[201], Mario Pirastu[202], Nicola Pirastu[58], Giorgio Pistis[203], Anna I. Podgornaia[10], Ozren Polasek[204,205], Belen Ponte[206], David J. Porteous[57, 207], Tanja Poulain[12], Peter P. Pramstaller[19], Michael H. Preuss[48], Bram P. Prins[55], Michael A. Province[5], Ton J. Rabelink[79,208], Laura M. Raffield[182], Olli T. Raitakari[209,210], Dermot F. Reilly[10], Rainer Rettig[211], Myriam Rheinberger[6], Kenneth M. Rice [43], Paul M. Ridker[108,212], Fernando Rivadeneira[25,213], Federica Rizzi[214,215], David J. Roberts[216], Antonietta Robino[21], Peter Rossing[31], Igor Rudan[58], Rico Rueedi[39,40], Daniela Ruggiero[18,71], Kathleen A.

Ryan[217], Yasaman Saba[218], Charumathi Sabanayagam[64], Veikko Salomaa[201], Erika Salvi[214,219], Kai-Uwe Saum[50], Helena Schmidt[220], Reinhold Schmidt[122], Ben Schöttker[50,51], Christina-Alexandra Schulz[32], Nicole Schupf[221,222,223], Christian M. Shaffer[59], Yuan Shi[64], Albert V. Smith[111], Blair H. Smith[224], Nicole Soranzo[225], Cassandra N. Spracklen[182], Konstantin Strauch[226,227], Heather M. Stringham[45], Michael Stumvoll[228], Per O. Svensson[229,230], Silke Szymczak[100], E-Shyong Tai[15,166,231], Salman M. Tajuddin[95], Nicholas Y. Q. Tan[64], Kent D. Taylor[232], Andrej Teren[12,233], Yih-Chung Tham[64], Joachim Thiery[12,53], Chris H. L. Thio[24], Hauke Thomsen[234], Gudmar Thorleifsson[16], Daniela Toniolo[74], Anke Tönjes[228], Johanne Tremblay[113,235], Ioanna Tzoulaki[90,236], André G. Uitterlinden[213], Simona Vaccargiu[202], Rob M. van Dam[15,166], Pim van der Harst[237,238,239], Cornelia M. van Duijn[25], Digna R. Velez Edwards[119,240], Niek Verweij[237], Suzanne Vogelezang[25,96,97], Uwe Völker[23,241], Peter Vollenweider[242,] Gerard Waeber[242], Melanie Waldenberger[105,106,147,] Lars Wallentin[243,244], Ya Xing Wang[138], Chaolong Wang[87, 245], Dawn M. Waterworth[4], Wen Bin Wei[246], Harvey White[247], John B. Whitfield[109], Sarah H. Wild[248], James F. Wilson[20,58], Mary K. Wojczynski[5], Charlene Wong[67], Tien-Yin Wong[64,67], Liang Xu[138,] Qiong Yang[192], Masayuki Yasuda[64,249], Laura M. Yerges-Armstrong[4], Weihua Zhang[61,90], Alan B. Zonderman[95], Jerome I. Rotter[232,250,251], Murielle Bochud[73], Bruce M. Psaty[252,253], Veronique Vitart[20], James G. Wilson[254], Abbas Dehghan[29,90], Afshin Parsa[255,256], Daniel I. Chasman[108,212], Kevin Ho[257,258], Andrew P. Morris[72,169], Olivier Devuyst[259], Shreeram Akilesh[37, 260], Sarah A. Pendergrass[261], Xueling Sim[15], Carsten A. Böger[6,262], Yukinori Okada[263,264], Todd L. Edwards[119,265], Harold Snieder[24], Kari Stefansson[16], Adriana M. Hung[119,266], Iris M. Heid[7], Markus Scholz[11,12], Alexander Teumer[22,23], Anna Köttgen[1,8] and Cristian Pattaro[19]

[1]Institute of Genetic Epidemiology, Department of Biometry, Epidemiology and Medical Bioinformatics, Faculty of Medicine and Medical Center–University of Freiburg, Freiburg, Germany. [2]Renal Division, Department of Medicine IV, Faculty of Medicine and Medical Center–University of Freiburg, Freiburg, Germany. [3]Division of Nephrology and Hypertension, Department of Medicine, University of Utah, Salt Lake City, USA. [4]Target Sciences–Genetics, GlaxoSmithKline, Collegeville, PA, USA. [5]Division of Statistical Genomics, Department of Genetics, Washington University School of Medicine, St. Louis, MO, USA. [6]Department of Nephrology, University Hospital Regensburg, Regensburg, Germany. [7]Department of Genetic Epidemiology, University of Regensburg, Regensburg, Germany. [8]Department of Epidemiology, Johns Hopkins Bloomberg School of Public Health, Baltimore, MD, USA. [9]Welch Center for Prevention, Epidemiology and Clinical Research, Baltimore, MD, USA. [10]Genetics, Merck & Co., Inc, Kenilworth, NJ, USA. [11]Institute for Medical Informatics, Statistics and Epidemiology, University of Leipzig, Leipzig, Germany. [12]LIFE Research Center for Civilization Diseases, University of Leipzig, Leipzig, Germany. [13]Division of Quantitative Sciences, Department of Obstetrics & Gynecology, Vanderbilt Genetics Institute, Vanderbilt Epidemiology Center, Institute for Medicine and Public Health, Vanderbilt University Medical Center, Nashville, TN, USA. [14]Biomedical Laboratory Research and Development, Tennessee Valley Healthcare System (626)/Vanderbilt University, Nashville, TN, USA. [15]Saw Swee Hock School of Public Health, National University of Singapore and National University Health System, Singapore, Singapore. [16]deCODE Genetics/Amgen, Inc., Reykjavik, Iceland. [17]Department of Public Health Sciences, Loyola University Chicago, Maywood, IL, USA. [18I]nstitute of Genetics and Biophysics 'Adriano Buzzati-Traverso'–CNR, Naples, Italy. [19]Eurac Research, Institute for Biomedicine (affiliated with the University of Lübeck), Bolzano, Italy. [20]Medical Research Council Human Genetics Unit, Institute of Genetics and Molecular Medicine, University of Edinburgh, Edinburgh, UK. [21]Institute for Maternal and Child Health, IRCCS 'Burlo Garofolo', Trieste, Italy. [22]Institute for

Community Medicine, University Medicine Greifswald, Greifswald, Germany. [23]DZHK (German Center for Cardiovascular Research), partner site Greifswald, Greifswald, Germany. [24]Department of Epidemiology, University of Groningen, University Medical Center Groningen, Groningen, the Netherlands. [25]Department of Epidemiology, Erasmus MC, University Medical Center Rotterdam, Rotterdam, the Netherlands. [26]Department of Biostatistics, Johns Hopkins Bloomberg School of Public Health, Baltimore, MD, USA. [27]Laboratory for Statistical Analysis, RIKEN Center for Integrative Medical Sciences (IMS), Yokohama, Japan. [28]Department of Ophthalmology, Graduate School of Medical Sciences, Kyushu University, Fukuoka, Japan. [29]Department of Epidemiology and Biostatistics, Faculty of Medicine, School of Public Health, Imperial College London, London, UK. [30]Institute of Public Health & Social Sciences, Khyber Medical University, Peshawar, Pakistan. [31]Steno Diabetes Center Copenhagen, Gentofte, Denmark. [32]Diabetes and Cardiovascular Disease–Genetic Epidemiology, Department of Clincial Sciences in Malmö, Lund University, Malmö, Sweden. [33]Division of Family Medicine and Primary Care, Department of Neurobiology, Care Sciences and Society, Karolinska Institutet, Stockholm, Sweden. [34]School of Health and Social Studies, Dalarna University, Stockholm, Sweden. [35]Division of Nephrology, Department of Internal Medicine, University of Groningen, University Medical Center Groningen, Groningen, the Netherlands. [36]Division of Nephrology, University of Washington, Seattle, WA, USA. [37]Kidney Research Institute, University of Washington, Seattle, WA, USA. [38]Cardiology, Geneva University Hospitals, Geneva, Switzerland. [39]Department of Computational Biology, University of Lausanne, Lausanne, Switzerland. [40]Swiss Institute of Bioinformatics, Lausanne, Switzerland. [41]Department of Integrative Biomedical Sciences, University of Cape Town, Cape Town, South Africa. [42]Cardiovascular Health Research Unit, Department of Medicine, University of Washington, Seattle, WA, USA. [43]Department of Biostatistics, University of Washington, Seattle, WA, USA. [44]Institute of Molecular Genetics, National Research Council of Italy, Pavia, Italy. [45]Department of Biostatistics and Center for Statistical Genetics, University of Michigan, Ann Arbor, MI, USA. [46]Human Genetics Center, University of Texas Health Science Center, Houston, TX, USA. [47]CNRS UMR 8199, European Genomic Institute for Diabetes (EGID), Institut Pasteur de Lille, University of Lille, Lille, France. [48]Charles Bronfman Institute for Personalized Medicine, Icahn School of Medicine at Mount Sinai, New York, NY, USA. [49]Digital Health Center, Hasso Plattner Institute and University of Potsdam, Potsdam, Germany. [50]Division of Clinical Epidemiology and Aging Research, German Cancer Research Center (DKFZ), Heidelberg, Germany. [51]Network Aging Research, University of Heidelberg, Heidelberg, Germany. [52]Department of Medicine, Surgery and Health Sciences, University of Trieste, Trieste, Italy. [53]Institute of Laboratory Medicine, Clinical Chemistry and Molecular Diagnostics, University of Leipzig, Leipzig, Germany. [54]Institute of Clinical Chemistry and Laboratory Medicine, University Hospital Regensburg, Regensburg, Germany. [55]MRC/BHF Cardiovascular Epidemiology Unit, Department of Public Health and Primary Care, University of Cambridge, Cambridge, UK. [56]National Institute for Health Research Blood and Transplant Research Unit in Donor Health and Genomics, University of Cambridge, Cambridge, UK. [57]Center for Genomic and Experimental Medicine, Institute of Genetics and Molecular Medicine, University of Edinburgh, Edinburgh, UK. [58]Center for Global Health Research, Usher Institute of Population Health Sciences and Informatics, University of Edinburgh, Edinburgh, UK. [59]Department of Biomedical Informatics, Vanderbilt University Medical Center, Nashville, TN, USA. [60]Lee Kong Chian School of Medicine, Nanyang Technological University, Singapore, Singapore. [61]Department of Cardiology, Ealing Hospital, Middlesex, UK. [62]Imperial College Healthcare NHS Trust, Imperial College London, London, UK. [63]MRC–PHE Center for Environment and Health, School of Public

Health, Imperial College London, London, UK. [64]Singapore Eye Research Institute, Singapore National Eye Center, Singapore, Singapore. [65]Department of Medical Epidemiology and Biostatistics, Karolinska Institutet, Stockholm, Sweden. [66]Ophthalmology and Visual Sciences Academic Clinical Program (Eye ACP), Duke–NUS Medical School, Singapore, Singapore. [67]Department of Ophthalmology, Yong Loo Lin School of Medicine, National University of Singapore and National University Health System, Singapore, Singapore. [68]Unit of Epidemiology, Biostatistics and Biodemography, Department of Public Health, Southern Denmark University, Odense, Denmark. [69]Center for Cardiovascular Prevention, Charles University in Prague, First Faculty of Medicine and Thomayer Hospital, Prague, Czech Republic. [70]Department of Medicine II, Charles University in Prague, First Faculty of Medicine, Prague, Czech Republic. [71]IRCCS Neuromed, Pozzilli, Italy. [72]Department of Biostatistics, University of Liverpool, Liverpool, UK. [73]Institute of Social and Preventive Medicine, Lausanne University Hospital, Lausanne, Switzerland. [74]San Raffaele Research Institute, Milan, Italy. [75]Institute of Biomedical Technologies, National Research Council of Italy, Milan, Italy. [76]Bio4Dreams–Business Nursery for Life Sciences, Milan, Italy. [77]Department of Public Health and Primary Care, School of Clinical Medicine, University of Cambridge, Cambridge, UK. [78]Department of Clinical Epidemiology, Leiden University Medical Center, Leiden, the Netherlands. [79]Section of Nephrology, Department of Internal Medicine, Leiden University Medical Center, Leiden, the Netherlands. [80]Institute of Clinical Molecular Biology, Christian-Albrechts-University of Kiel, Kiel, Germany. [81]Department of Medicine (Nephrology, Hypertensiology, Rheumatology, Endocrinology, Diabetology), Medical Faculty Mannheim, University of Heidelberg, Mannheim, Germany. [82]Department of Public Health and Primary Care, University of Cambridge, Cambridge, UK. [83]NHS Blood and Transplant, Cambridge, UK. [84]Department of Women and Child Health, Hospital for Children and Adolescents, University of Leipzig, Leipzig, Germany. [85]Center for Pediatric Research, University of Leipzig, Leipzig, Germany. [86]Public Health Sciences–Biostatistics, Wake Forest School of Medicine, Winston-Salem, NC, USA. [87]Genome Institute of Singapore, Agency for Science Technology and Research, Singapore, Singapore. [88]Intensive Care Medicine, Charité, Berlin, Germany. [89]Department of Nephrology and Hypertension, Friedrich Alexander University Erlangen-Nürnberg (FAU), Erlangen, Germany. [90]Department of Epidemiology and Biostatistics, MRC-PHE Center for Environment and Health, School of Public Health, Imperial College London, London, UK. [91]Imperial College NIHR Biomedical Research Center, Imperial College London, London, UK. [92]Dementia Research Institute, Imperial College London, London, UK. [93]Health Data Research UK–London, London, UK. [94]Department of Anatomy and Cell Biology, University Medicine Greifswald, Greifswald, Germany. [95]Laboratory of Epidemiology and Population Sciences, National Institute on Aging, Intramural Research Program, US National Institutes of Health, Baltimore, MD, USA. [96]Generation R Study Group, Erasmus MC, University Medical Center Rotterdam, Rotterdam, the Netherlands. [97]Department of Pediatrics, Erasmus MC, University Medical Center Rotterdam, Rotterdam, The Netherlands. [98]Institute of Social and Preventive Medicine (ISPM), University of Bern, Bern, Switzerland. [99]Section on Nephrology, Internal Medicine, Wake Forest School of Medicine, Winston-Salem, NC, USA. [100]Institute of Medical Informatics and Statistics, Kiel University, University Hospital Schleswig-Holstein, Kiel, Germany. [101]School of Public Health and Community Medicine, Hebrew University of Jerusalem, Jerusalem, Israel. [102]Department of Genomics of Common Disease, Imperial College London, London, UK. [103]Massachusetts Veterans Epidemiology Research and Information Center, VA Cooperative Studies Program, VA Boston Healthcare System, Boston, MA, USA. [104]Molecular Geriatrics, Department of Public Health and Caring Sciences, Uppsala

University, Uppsala, Sweden. [105]Research Unit of Molecular Epidemiology, Helmholtz Zentrum München–German Research Center for Environmental Health, Neuherberg, Germany. [106]Institute of Epidemiology, Helmholtz Zentrum München—German Research Center for Environmental Health, Neuherberg, Germany. [107]German Center for Diabetes Research (DZD), Neuherberg, Germany. [108]Division of Preventive Medicine, Brigham and Women's Hospital, Boston, MA, USA. [109]QIMR Berghofer Medical Research Institute, Brisbane, Queensland, Australia. [110]Icelandic Heart Association, Kopavogur, Iceland. [111]Faculty of Medicine, School of Health Sciences, University of Iceland, Reykjavik, Iceland. [112]Estonian Genome Center, Institute of Genomics, University of Tartu, Tartu, Estonia. [113]Montreal University Hospital Research Center, CHUM, Montreal, Quebec, Canada. [114]Medpharmgene, Montreal, Quebec, Canada. [115]Laboratory of Epidemiology and Population Sciences, National Institute on Aging, Intramural Research Program, US National Institutes of Health, Bethesda, MD, USA. [116]Interdisciplinary Center of Psychopathology and Emotion Regulation (ICPE), University of Groningen, University Medical Center Groningen, Groningen, the Netherlands. [117]Vanderbilt Genetics Institute, Vanderbilt University Medical Center, Nashville, TN, USA. [118]Division of Epidemiology, Department of Medicine, Vanderbilt Genetics Institute, Vanderbilt University Medical Center, Nashville, TN, USA. [119]Department of Veteran's Affairs, Tennessee Valley Healthcare System (626)/Vanderbilt University, Nashville, TN, USA. [120]Department of Paediatrics, Yong Loo Lin School of Medicine, National University of Singapore, Singapore, Singapore. [121]Khoo Teck Puat–National University Children's Medical Institute, National University Health System, Singapore, Singapore. [122]Clinical Division of Neurogeriatrics, Department of Neurology, Medical University of Graz, Graz, Austria. [123]Institute for Medical Informatics, Statistics and Documentation, Medical University of Graz, Graz, Austria. [124]Department of Genetics, Shanghai–MOST Key Laboratory of Health and Disease Genomics, Chinese National Human Genome Center, Shanghai, China. [125]Shanghai Industrial Technology Institute, Shanghai, China. [126]Department of Pediatrics, Tampere University Hospital, Tampere, Finland. [127]Department of Pediatrics, Faculty of Medicine and Life Sciences, University of Tampere, Tampere, Finland. [128]NHLBI's Framingham Heart Study, Framingham, MA, USA. [129]The Center for Population Studies, NHLBI, Framingham, MA, USA. [130]Division of Nephrology, Internal Medicine Services, Landspitali–The National University Hospital of Iceland, Reykjavik, Iceland. [131]Division of Cardiovascular Medicine, Department of Medicine, Stanford University School of Medicine, Stanford, CA, USA. [132]Stanford Cardiovascular Institute, Stanford University, Stanford, CA, USA. [133]Molecular Epidemiology and Science for Life Laboratory, Department of Medical Sciences, Uppsala University, Uppsala, Sweden. [134]Stanford Diabetes Research Center, Stanford University, Stanford, CA, USA. [135]Max Planck Institute of Psychiatry, Munich, Germany. [136]The Center of Public Health Sciences, University of Iceland, Reykjavík, Iceland. [137]Department of Ophthalmology, Medical Faculty Mannheim, University Heidelberg, Mannheim, Germany. [138]Beijing Institute of Ophthalmology, Beijing Key Laboratory of Ophthalmology and Visual Sciences, Beijing Tongren Hospital, Capital Medical University, Beijing, China. [139]Geisinger Research, Biomedical and Translational Informatics Institute, Rockville, MD, USA. [140]Department of Clinical Physiology, Tampere University Hospital, Tampere, Finland. [141]Department of Clinical Physiology, Finnish Cardiovascular Research Center–Tampere, Faculty of Medicine and Health Technology, Tampere University, Tampere, Finland. [142]Kyoto-McGill International Collaborative School in Genomic Medicine, Kyoto University Graduate School of Medicine, Kyoto, Japan. [143]Department of Human Genetics, Graduate School of Public Health, University of Pittsburgh, Pittsburgh, PA, USA. [144]Department of Biomedical Informatics, Harvard Medical School, Boston, MA, USA. [145]Department of Medicine, Kuopio

University Hospital, Kuopio, Finland. [146]Deutsches Herzzentrum München, Technische Universität München, Munich, Germany. [147]DZHK (German Center for Cardiovascular Research), Partner Site Munich Heart Alliance, Munich, Germany. [148]Institute of Epidemiology and Biostatistics, University of Ulm, Ulm, Germany. [149]National Heart and Lung Institute, Imperial College London, London, UK. [150]Integrated Research and Treatment Center Adiposity Diseases, University of Leipzig, Leipzig, Germany. [151]Division of Nephrology and Hypertension, Loyola University Chicago, Chicago, IL, USA. [152]Division of Genetic Epidemiology, Department of Medical Genetics, Molecular and Clinical Pharmacology, Medical University of Innsbruck, Innsbruck, Austria. [153]RIKEN Center for Integrative Medical Sciences (IMS), Yokohama (Kanagawa), Japan. [154]The Department of Public Health Solutions, National Institute for Health and Welfare, Helsinki, Finland. [155]Diabetes and Obesity Research Program, University of Helsinki, Helsinki, Finland. [156]Institute of Clinical Medicine, Internal Medicine, University of Eastern Finland, Kuopio, Finland. [157]Division of Biomedical Informatics and Personalized Medicine, School of Medicine, University of Colorado Denver–Anschutz Medical Campus, Aurora, CO, USA. [158]Department of Clinical Chemistry, Fimlab Laboratories, Tampere, Finland. [159]Department of Clinical Chemistry, Finnish Cardiovascular Research Center–Tampere, Faculty of Medicine and Life Sciences, University of Tampere, Tampere, Finland. [160]Institute of Epidemiology and Biobank Popgen, Kiel University, Kiel, Germany. [161]A list of members and affiliations appears in the Supplementary Note available at https://static-content.springer.com/esm/art%3A10.1038%2Fs41588-019-0407-x/MediaObjects/41588_2019_407_MOESM1_ESM.pdf. [162]Diabetes Center, Khoo Teck Puat Hospital, Singapore, Singapore. [163]Cardiovascular Epidemiology, Department of Medical Sciences, Uppsala University, Uppsala, Sweden. [164]Nuffield Department of Medicine, University of Oxford, Oxford, UK. [165]Broad Institute of Harvard and MIT, Cambridge, MA, USA. [166]Department of Medicine, Yong Loo Lin School of Medicine, National University of Singapore and National University Health System, Singapore, Singapore. [167]The Mindich Child Health and Development Institute, Icahn School of Medicine at Mount Sinai, New York, NY, USA. [168]Target Sciences—Genetics, GlaxoSmithKline, Albuquerque, NM, USA. [169]Wellcome Trust Center for Human Genetics, University of Oxford, Oxford, UK. [170]Oxford Center for Diabetes, Endocrinology and Metabolism, University of Oxford, Oxford, UK. [171]Department of Translational Research in Psychiatry, Max Planck Institute of Psychiatry, Munich, Germany. [172]Synlab Academy, Synlab Holding Deutschland GmbH, Mannheim, Germany. [173]Clinical Institute of Medical and Chemical Laboratory Diagnostics, Medical University of Graz, Graz, Austria. [174]Medical Clinic V, Medical Faculty Mannheim, University of Heidelberg, Mannheim, Germany. [175]Laboratory of Clinical Genome Sequencing, Graduate School of Frontier Sciences, The University of Tokyo, Tokyo, Japan. [176]Independent Research Group Clinical Epidemiology, Helmholtz Zentrum München, German Research Center for Environmental Health, Neuherberg, Germany. [177]Chair of Epidemiology, Ludwig- Maximilians-Universität München at UNIKA-T Augsburg, Augsburg, Germany. [178]Institute of Human Genetics, Helmholtz Zentrum München, Neuherberg, Germany. [179]Institute of Human Genetics, Technische Universität München, Munich, Germany. [180]Hypertension and Cardiovascular Disease, Department of Clincial Sciences Malmö, Lund University, Malmö, Sweden. [181]Department of Psychiatry, VU University Medical Center, Amsterdam, the Netherlands. [182]Department of Genetics, University of North Carolina, Chapel Hill, NC, USA. [183]Institute for Molecular Bioscience, University of Queensland, St Lucia, Queensland, Australia. [184]Department of Public Health and Primary Care, Leiden University Medical Center, Leiden, the Netherlands. [185]Center for Public Health Genomics, University of Virginia, Charlottesville, Charlottesville, VA, USA. [186]Division of Nephrology,

Department of Medicine, Icahn School of Medicine at Mount Sinai, New York, NY, USA. [187]Laboratory of Neurogenetics, National Institute on Aging, National Institutes of Health, Bethesda, MD, USA. [188]Data Tecnica International, Glen Echo, MD, USA. [189]Institute of Clinical Chemistry and Laboratory Medicine, University Medicine Greifswald, Greifswald, Germany. [190]Department of Cardiology, Heart Center, Tampere University Hospital, Tampere, Finland. [191]Department of Cardiology, Finnish Cardiovascular Research Center–Tampere, Faculty of Medicine and Life Sciences, Tampere University, Tampere, Finland. [192]Department of Biostatistics, Boston University School of Public Health, Boston, MA, USA. [193]Section of Gerontology and Geriatrics, Department of Internal Medicine, Leiden University Medical Center, Leiden, the Netherlands. [194]University of Maryland School of Medicine, Baltimore, MD, USA. [195]Cardiovascular Division, Brigham and Women's Hospital, Boston, MA, USA. [196]TIMI Study Group, Boston, MA, USA. [197]Department of Clinical Biochemistry, Landspitali University Hospital, Reykjavik, Iceland. [198]Institute of Cardiovascular and Medical Sciences, University of Glasgow, Glasgow, UK. [199]Biochemistry, Wake Forest School of Medicine, Winston-Salem, NC, USA. [200]Department of Medicine, Geriatrics Section, Boston Medical Center, Boston University School of Medicine, Boston, MA, USA. [201]National Institute for Health and Welfare, Helsinki, Finland. [202]Institute of Genetic and Biomedical Research, National Research Council of Italy, UOS of Sassari, Li Punti, Sassari, Italy. [203]Department of Psychiatry, University Hospital of Lausanne, Lausanne, Switzerland. [204]Faculty of Medicine, University of Split, Split, Croatia. [205]Gen-info Ltd, Zagreb, Croatia. [206]Service de Néphrologie, Geneva University Hospitals, Geneva, Switzerland. [207]Center for Cognitive Ageing and Cognitive Epidemiology, University of Edinburgh, Edinburgh, UK. [208]Einthoven Laboratory of Experimental Vascular Research, Leiden University Medical Center, Leiden, the Netherlands. [209]Department of Clinical Physiology and Nuclear Medicine, Turku University Hospital, Turku, Finland. [210]Research Center of Applied and Preventive Cardiovascular Medicine, University of Turku, Turku, Finland. [211]Institute of Physiology, University Medicine Greifswald, Karlsburg, Germany. [212]Harvard Medical School, Boston, MA, USA. [213]Department of Internal Medicine, Erasmus MC, University Medical Center Rotterdam, Rotterdam, the Netherlands. [214]Department of Health Sciences, University of Milan, Milano, Italy. [215]ePhood Scientific Unit, ePhood SRL, Milano, Italy. [216]NHS Blood and Transplant, BRC Oxford Haematology Theme; Nuffield Division of Clinical Laboratory Sciences; University of Oxford, Oxford, UK. [217]Division of Endocrinology, Diabetes and Nutrition, University of Maryland School of Medicine, Baltimore, MD, USA. [218]Molecular Biology and Biochemistry, Gottfried Schatz Research Center for Cell Signaling, Metabolism and Aging, Medical University of Graz, Graz, Austria. [219]Neuroalgology Unit, Fondazione IRCCS Istituto Neurologico 'Carlo Besta', Milan, Italy. [220]Institute of Molecular Biology and Biochemistry, Center for Molecular Medicine, Medical University of Graz, Graz, Austria. [221]Department of Neurology, College of Physicians and Surgeons, Columbia University, New York, NY, USA. [222]Gertrude H. Sergievsky Center, Columbia University Medical Center, New York, NY, USA. [223]Taub Institute for Research on Alzheimer's Disease and the Aging Brain, Columbia University Medical Center, New York, USA. [224]Division of Population Health and Genomics, Ninewells Hospital and Medical School, University of Dundee, Dundee, UK. [225]Human Genetics, Wellcome Sanger Institute, Hinxton, UK. [226]Institute of Genetic Epidemiology, Helmholtz Zentrum München—German Research Center for Environmental Health, Neuherberg, Germany. [227]Chair of Genetic Epidemiology, IBE, Faculty of Medicine, Ludwig-Maximilians-Universität München, München, Germany. [228]Department of Endocrinology and Nephrology, University of Leipzig, Leipzig, Germany. [229]Department of Clinical Science and Education, Karolinska Institutet, Södersjukhuset, Stockholm, Sweden. [230]Department of Cardiology, Södersjukhuset, Stockholm,

Sweden. [231]Duke–NUS Medical School, Singapore, Singapore. [232]The Institute for Translational Genomics and Population Sciences, Department of Pediatrics, Los Angeles Biomedical Research Institute at Harbor–UCLA Medical Center, Torrance, CA, USA. [233]Heart Center Leipzig, Leipzig, Germany. [234]Division of Molecular Genetic Epidemiology, German Cancer Research Center (DKFZ), Heidelberg, Germany. [235]CRCHUM, Montreal, Canada. [236]Department of Hygiene and Epidemiology, University of Ioannina Medical School, Ioannina, Greece. [237]Department of Cardiology, University of Groningen, University Medical Center Groningen, Groningen, the Netherlands. [238]Department of Genetics, University of Groningen, University Medical Center Groningen, Groningen, the Netherlands. [239]Durrer Center for Cardiovascular Research, The Netherlands Heart Institute, Utrecht, the Netherlands. [240]Department of Obstetrics and Gynecology, Institute for Medicine and Public Health, Vanderbilt University Medical Center, Nashville, TN, USA. [241]Interfaculty Institute for Genetics and Functional Genomics, University Medicine Greifswald, Greifswald, Germany. [242]Internal Medicine, Department of Medicine, Lausanne University Hospital, Lausanne, Switzerland. [243]Cardiology, Department of Medical Sciences, Uppsala University, Uppsala, Sweden. [244]Uppsala Clinical Research Center, Uppsala University, Uppsala, Sweden. [245]School of Public Health, Tongji Medical College, Huazhong University of Science and Technology, Wuhan, China. [246]Beijing Tongren Eye Center, Beijing Tongren Hospital, Capital Medical University, Beijing, China. [247]Green Lane Cardiovascular Service, Auckland City Hospital and University of Auckland, Auckland, New Zealand. [248]Center for Population Health Sciences, Usher Institute of Population Health Sciences and Informatics, University of Edinburgh, Edinburgh, UK. [249]Department of Ophthalmology, Tohoku University Graduate School of Medicine, Miyagi, Japan. [250]Department of Pediatrics, Harbor–UCLA Medical Center, Torrance, CA, USA. [251]Department of Medicine, Harbor–UCLA Medical Center, Torrance, CA, USA. [252]Cardiovascular Health Research Unit, Department of Medicine, Department of Epidemiology, Department of Health Service, University of Washington, Seattle, WA, USA. [253]Kaiser Permanente Washington Health Research Institute, Seattle, WA, USA. [254]Department of Physiology and Biophysics, University of Mississippi Medical Center, Jackson, MS, USA. [255]Division of Kidney, Urologic and Hematologic Diseases, National Institute of Diabetes and Digestive and Kidney Diseases, National Institutes of Health, Bethesda, MD, USA. [256]Department of Medicine, University of Maryland School of Medicine, Baltimore, MD, USA. [257]Kidney Health Research Institute (KHRI), Geisinger, Danville, PA, USA. [258]Department of Nephrology, Geisinger, Danville, PA, USA. [259]Institute of Physiology, University of Zurich, Zurich, Switzerland. [260]Anatomic Pathology, University of Washington Medical Center, Seattle, WA, USA. [261]Geisinger Research, Biomedical and Translational Informatics Institute, Danville, PA, USA. [262]Department of Nephrology and Rheumatology, Kliniken Südostbayern, Regensburg, Germany. [263]Laboratory for Statistical Analysis, RIKEN Center for Integrative Medical Sciences (IMS), Osaka, Japan. [264]Department of Statistical Genetics, Osaka University Graduate School of Medicine, Osaka, Japan. [265]Division of Epidemiology, Department of Medicine, Vanderbilt Genetics Institute, Vanderbilt University Medical Center, Nashville, TN, USA. [266]Vanderbilt University Medical Center, Division of Nephrology & Hypertension, Nashville, TN, USA.

Supplementary References

1. Gorski, M. *et al.* 1000 Genomes-based meta-analysis identifies 10 novel loci for kidney function. *Sci Rep* **7**, 45040 (2017).

2. Pattaro, C. *et al.* Genetic associations at 53 loci highlight cell types and biological pathways relevant for kidney function. *Nat Commun* **7**, 10023 (2016).
3. Pers, T.H. *et al.* Biological interpretation of genome-wide association studies using predicted gene functions. *Nat Commun* **6**, 5890 (2015).
