# Supplementary material for: Mapping eGFR loci to the renal transcriptome and phenome in the VA Million Veteran Program
Source: Nat Commun. 2019 Aug 26;10:3842. doi: 10.1038/s41467-019-11704-w (PMC6710266; doi:10.1038/s41467-019-11704-w)
Supplement: Supplementary file 11 — Description of Additional Supplementary Files [file 41467_2019_11704_MOESM11_ESM.docx]

**Title: Supplementary Data 1**. **Association results for all significant variants from trans-ethnic analysis of eGFR.
Description:** SNPs are ordered by tier of replication, then by chromosome and position. rsID - dbSNP accession number; CHR:BP - chromosome and build 37 position; Nearest Gene - most proximal gene within 250kb of index SNP; Distance - distance in base pairs from index SNP to nearest gene; Location - location of index SNP relative to nearest gene; Effect allele - allele corresponding to measured effect on the outcome; Other allele - allele not corresponding to measured effect on the outcome; Novelty eGFR – annotation of whether locus is novel or previously identified in a GWAS of eGFR; Tier - evidentiary tier for replicated association 1) Genome-wide significance in the discovery stage, and Genome-wide significance in the replication stage, 2) Genome-wide significance in the discovery stage, and 5x10-8 < p ≤ Bonferroni correction for 122 SNPs = 0.0004 in replication and consistent trait-specific direction of effect across stages, or 3) Genome-wide significance in the discovery stage, and 0.0004 < p ≤ 0.05 in the replication stage and consistent trait-specific direction of effect across stages; EAF_disc_ - effect allele frequency in the discovery set; Effect_disc_ - measured effect in the discovery meta-analysis; SE_disc_ - standard error of the measured effect in the discovery meta-analysis; P-value_disc_ - association p-value for the measured effect in the discovery meta-analysis; Effect_rep_ - measured effect in the replication meta-analysis; SE_rep_ - standard error of the measured effect in the replication meta-analysis; P-value_rep_ - association p-value for the measured effect in the replication meta-analysis.

**Title: Supplementary Data 2**. **Association results for all eGFR replicated variants from whites-only analysis.
Description:** SNPs are ordered by tier of replication, then by chromosome and position. rsID - dbSNP accession number; CHR:BP - chromosome and build 37 position; Nearest Gene - most proximal gene within 250kb of index SNP; Distance - distance in base pairs from index SNP to nearest gene; Location - location of index SNP relative to nearest gene; Effect allele - allele corresponding to measured effect on the outcome; Other allele - allele not corresponding to measured effect on the outcome; Novelty eGFR – annotation of whether locus is novel or previously identified in a GWAS of eGFR; Tier - evidentiary tier for replicated association 1) Genome-wide significance in the discovery stage, and Genome-wide significance in the replication stage, 2) Genome-wide significance in the discovery stage, and 5x10-8 < p ≤ Bonferroni correction for 122 SNPs = 0.0004 in replication and consistent trait-specific direction of effect across stages, or 3) Genome-wide significance in the discovery stage, and 0.0004 < p ≤ 0.05 in the replication stage and consistent trait-specific direction of effect across stages; EAF_disc_ - effect allele frequency in the combined discovery and replication meta-analysis; Effect_disc_ - measured effect in the discovery meta-analysis; SE_disc_ - standard error of the measured effect in the discovery meta-analysis; P-value_disc_ - association p-value for the measured effect in the discovery meta-analysis; Effect_rep_ - measured effect in the replication meta-analysis; SE_rep_ - standard error of the measured effect in the replication meta-analysis; P-value_rep_ - association p-value for the measured effect in the replication meta-analysis.

**Title: Supplementary Data 3**. **Association results for significant eGFR results in DM.
Description:** SNPs are ordered by p-value. rsID - dbSNP accession number; CHR:BP - chromosome and build 37 position; Effect allele - allele corresponding to measured effect on the outcome; Other allele - allele not corresponding to measured effect on the outcome; EAF - effect allele frequency in the discovery meta-analysis; Effect - measured effect in the discovery meta-analysis; SE - standard error of the measured effect in the discovery meta-analysis; P-value - association p-value for the measured effect in the discovery meta-analysis; Nearest Gene - most proximal gene within 250kb of index SNP; Distance - distance in base pairs from index SNP to nearest gene.

**Title: Supplementary Data 4**. **Association results for significant eGFR results in nonDM.
Description:** SNPs are ordered by p-value. rsID - dbSNP accession number; CHR:BP - chromosome and build 37 position; Effect allele - allele corresponding to measured effect on the outcome; Other allele - allele not corresponding to measured effect on the outcome; EAF - effect allele frequency in the discovery meta-analysis; Effect - measured effect in the discovery meta-analysis; SE - standard error of the measured effect in the discovery meta-analysis; P-value - association p-value for the measured effect in the discovery meta-analysis; Nearest Gene - most proximal gene within 250kb of index SNP; Distance - distance in base pairs from index SNP to nearest gene.

**Title: Supplementary Data 5**. **Significant human kidney S-PrediXcan results for transethnic eGFR and stratified by diabetes status.**
**Description:** Table is sorted by p-value in overall eGFR results. Gene - gene name from the transcriptome model mapped to ensembl genes, generally extracted from Genquant; Z-score - S-PrediXcan's association result for the gene; Effect - S-PrediXcan 's association effect size for the gene; P-value - P-value of the aforementioned statistic; var_g - variance of the gene expression, calculated as W' * G * W (where W is the vector of SNP weights in a gene's model, W' is its transpose, and G is the covariance matrix); pred_perf_r2: R2 of tissue model's correlation to gene's measured transcriptome (prediction performance); pred_perf_pval: pval of tissue model's correlation to gene's measured transcriptome (prediction performance); pred_perf_qval: qval of tissue model's correlation to gene's measured transcriptome (prediction performance); N_SNPs_used: number of SNPs from GWAS that got used in S-PrediXcan analysis; N_SNPs_inModel: number of SNPs in the model;

**Title: Supplementary Data 6. DEPICT tissue enrichment results across all significant (P<5x10-8) eGFR GWAS loci from transethnic analysis of all subjects.
Description:** Table is sorted by p-value. MeSH Tree Numbers - Medical Subject Heading (MeSH) tissue and cell type annotations for which genes are highly expressed; MeSH first level term - name of the most specific term in the MeSH Tree Number; MeSH second level term - name of the least specific term in the MeSH Tree Number; P - nominal p-value for enrichment; FDR < 5% - yes/no indicator of whether the false discovery rate q-value was less than 5%.

**Title: Supplementary Data 7. DEPICT tissue enrichment results across all significant (P<5x10-8) eGFR GWAS loci from transethnic analysis of subjects without diabetes.
Description:** Table is sorted by p-value. MeSH Tree Numbers - Medical Subject Heading (MeSH) tissue and cell type annotations for which genes are highly expressed; MeSH first level term - name of the most specific term in the MeSH Tree Number; MeSH second level term - name of the least specific term in the MeSH Tree Number; P - nominal p-value for enrichment; FDR < 5% - yes/no indicator of whether the false discovery rate q-value was less than 5%.

**Title: Supplementary Data 8. DEPICT gene set enrichment results across all significant (P<5x10-8) eGFR GWAS loci from transethnic analysis of all subjects.**
**Description:** Table is sorted by p-value. Original gene set ID - name of DEPICT gene set tested for enrichment; Original gene set description - description of DEPICT gene set tested for enrichment; P - nominal p-value for enrichment; FDR < 5% - yes/no indicator of whether the false discovery rate q-value was less than 5%.

**Title: Supplementary Data 9. DEPICT gene set enrichment results across all significant (P<5x10-8) eGFR GWAS loci from transethnic analysis of subjects without diabetes.**
Table is sorted by p-value. Original gene set ID - name of DEPICT gene set tested for enrichment; Original gene set description - description of DEPICT gene set tested for enrichment; P - nominal p-value for enrichment; FDR < 5% - yes/no indicator of whether the false discovery rate q-value was less than 5%.
