# Supplementary material for: Mapping eGFR loci to the renal transcriptome and phenome in the VA Million Veteran Program
Source: Nat Commun. 2019 Aug 26;10:3842. doi: 10.1038/s41467-019-11704-w (PMC6710266; doi:10.1038/s41467-019-11704-w)
Supplement: Supplementary file 12 — Reporting Summary [file 41467_2019_11704_MOESM12_ESM.pdf]

nature research

Corresponding author(s): Todd L. Edwards, Adriana M. Hung

Last updated by author(s): 06/28/2019

Reporting Summary

Nature Research wishes to improve the reproducibility of the work that we publish. This form provides structure for consistency and transparency in reporting. For further information on Nature Research policies, see Authors & Referees and the Editorial Policy Checklist.

Statistics

For all statistical analyses, confirm that the following items are present in the figure legend, table legend, main text, or Methods section.

n/a	Confirmed	
☐	☒	The exact sample size (*n*) for each experimental group/condition, given as a discrete number and unit of measurement
☐	☒	A statement on whether measurements were taken from distinct samples or whether the same sample was measured repeatedly
☐	☒	The statistical test(s) used AND whether they are one- or two-sided<br>*Only common tests should be described solely by name; describe more complex techniques in the Methods section.*
☐	☒	A description of all covariates tested
☐	☒	A description of any assumptions or corrections, such as tests of normality and adjustment for multiple comparisons
☐	☒	A full description of the statistical parameters including central tendency (e.g. means) or other basic estimates (e.g. regression coefficient) AND variation (e.g. standard deviation) or associated estimates of uncertainty (e.g. confidence intervals)
☐	☒	For null hypothesis testing, the test statistic (e.g. *F*, *t*, *r*) with confidence intervals, effect sizes, degrees of freedom and *P* value noted<br>*Give P values as exact values whenever suitable.*
☒	☐	For Bayesian analysis, information on the choice of priors and Markov chain Monte Carlo settings
☒	☐	For hierarchical and complex designs, identification of the appropriate level for tests and full reporting of outcomes
☐	☒	Estimates of effect sizes (e.g. Cohen's *d*, Pearson's *r*), indicating how they were calculated

Our web collection on statistics for biologists contains articles on many of the points above.

Software and code

Policy information about availability of computer code

Data collection

NA; data were already collected and de-identified and provided for researchers to use

Data analysis

Several data analysis software programs were used for this study:

URLs:
Affymetrix Power Tools 2.10.0: https://www.thermofisher.com/us/en/home/life-science/microarray-analysis/affymetrix.html
BIDD TTD database: https://db.idrblab.org/ttd/
Corporate Data Warehouse: https://www.hsrd.research.va.gov/for_researchers/vinci/cdw.cfm
DEPICT: https://data.broadinstitute.org/mpg/depict/
EAGLE v2: https://data.broadinstitute.org/alkesgroup/Eagle/
Ensembl BioMart: http://www.ensembl.org/biomart/martview
FlashPCA2: https://github.com/gabraham/flashpca
GCTA v1.91.4beta: http://cnsgenomics.com/software/gcta/#Overview
GTEx portal: https://www.gtexportal.org/home/
GWAS catalog: https://www.ebi.ac.uk/gwas/
Human Protein Atlas: https://www.proteinatlas.org/
KING software:http://people.virginia.edu/~wc9c/KING/
LDSC v1.0.0: https://github.com/bulik/ldsc
METAL software: http://csg.sph.umich.edu/abecasis/metal/
Minimac3 : https://genome.sph.umich.edu/wiki/Minimac3
Observational Medical Outcomes Partnership: https://fnih.org/what-we-do/major-completed-programs/omop
PheWAS package: https://github.com/PheWAS/PheWAS
R statistical software: https://www.r-project.org/
SNPDOC: https://wakegen.phs.wakehealth.edu/public/snpdoc3/index.cfm

SNPTEST-v2.5.4-beta: https://mathgen.stats.ox.ac.uk/genetics_software/snptest/old/snptest_v2.3.0.html
S-PrediXcan: https://github.com/hakyimlab/MetaXcan

For manuscripts utilizing custom algorithms or software that are central to the research but not yet described in published literature, software must be made available to editors/reviewers. We strongly encourage code deposition in a community repository (e.g. GitHub). See the Nature Research guidelines for submitting code & software for further information.

Data

Policy information about availability of data

All manuscripts must include a data availability statement. This statement should provide the following information, where applicable:

- Accession codes, unique identifiers, or web links for publicly available datasets
- A list of figures that have associated raw data
- A description of any restrictions on data availability

Full summary statistics relating to the Million Veteran Program (MVP) studies are available at dbGAP accession phs001672.v2.p1 [https://www.ncbi.nlm.nih.gov/projects/gap/cgi-bin/study.cgi?study_id=phs001672.v2.p1]. Statistically significant reports for S-PrediXcan results for human kidney tissues and PheWAS analyses for eGFR are made available in the supplementary data and tables.

Field-specific reporting

Please select the one below that is the best fit for your research. If you are not sure, read the appropriate sections before making your selection.

☒ Life sciences ☐ Behavioural & social sciences ☐ Ecological, evolutionary & environmental sciences

For a reference copy of the document with all sections, see nature.com/documents/nr-reporting-summary-flat.pdf

Life sciences study design

All studies must disclose on these points even when the disclosure is negative.

Sample size	These sample sizes were chosen based on availability of primary data from the Million Veteran Program. With a total of >280,000 individuals for discovery only followed up by availability of over 700,000 through CKDGen for replication, we were confident that these available sample sizes would be sufficient to detect many novel genetic variants associated with eGFR not detected before.
Data exclusions	Data points that were extreme outliers (i.e. Scr values less than 0.4mg/dl) were excluded. Within pairs of individuals with cryptic relatedness (halfway between 2nd and 3rd degree relatives or closer), one of the two were excluded from analysis in order to maintain assumption of independent individuals in the study.
Replication	Index genetic variants from independent loci (within 500kb from the peak and r2<0.1) presenting with statistical significance (P-value < 5E-8) and up to two proxy SNPs for locus were further investigated for replication using data from the CKDGen consortium.
Randomization	This is an observational study.
Blinding	Blinding does not apply here as this is an observational study where the exposures of interest (genetic variants are not modifiable).

Reporting for specific materials, systems and methods

We require information from authors about some types of materials, experimental systems and methods used in many studies. Here, indicate whether each material, system or method listed is relevant to your study. If you are not sure if a list item applies to your research, read the appropriate section before selecting a response.

Materials & experimental systems

n/a	Involved in the study	
☒	☐	Antibodies
☒	☐	Eukaryotic cell lines
☒	☐	Palaeontology
☐	☒	Animals and other organisms
☐	☒	Human research participants
☒	☐	Clinical data

Methods

n/a	Involved in the study	
☒	☐	ChIP-seq
☒	☐	Flow cytometry
☒	☐	MRI-based neuroimaging

Animals and other organisms

Policy information about studies involving animals; ARRIVE guidelines recommended for reporting animal research

Laboratory animals	Data generated from animals for this study were generated and described previously (https://www.ncbi.nlm.nih.gov/pubmed/29622724). Animal studies were approved by the Institutional Animal Care and Use Committee (IACUC) of the

University of Pennsylvania. We mated Cdh16Cre mice (Jackson Lab, 012237), Nphs2Cre mice (Jackson Lab, 008205), SclCre mice (MGI number is 3579158) Aqp2Cre mice (Jackson Lab, 006881) Atp6v1b1Cre (15) mice with Tomato-GFP (mT/mG) mice (Jackson Lab, 007576) to generate Cdh16CremT/mG, SclCremT/mG, Nphs2cremT/mG mice, Atp6v1b1CremT/mG, Aqp2CremT/mG animals. The SclCremT/mG mice was injected with tamoxifen (40 mg/kg) three consecutive days and kidneys were harvested 2 weeks later. Unless otherwise noted 4-8 weeks of old male C57BL/6 mice were used as controls. Pax8rtTA/TRENICD animals were generated by mating the NICD mice (32) with Pax8-rtTA (Jackson Lab, 007176). Subsequently, the mice were placed on doxycycline-containing chow for 1 week starting at 4 weeks of age. Genotyping was confirmed by tail PCR using published primers.

Wild animals

No wild animals were used in this study.

Field-collected samples

No field-collected samples were used in this study.

Ethics oversight

Animal studies were approved by the Institutional Animal Care and Use Committee (IACUC) of the University of Pennsylvania.

Note that full information on the approval of the study protocol must also be provided in the manuscript.

Human research participants

Policy information about studies involving human research participants

Population characteristics

MVP participants (N=280,722),representing the discovery sample size,were predominantly male (93%),and were administratively identified as non-Hispanic white (80%) or non-Hispanic black (20%)(Supplementary Table1). When evaluating the data stratified by diabetes status, there were more diabetics than non-diabetics who were hypertensive within both race groups (non-Hispanic white diabetics and hypertensive 91%; non-Hispanic black diabetics and hypertensive 93%). Across both race groups, eGFR was lower in diabetics than in non-diabetics, and eGFR was higher in non-Hispanic blacks than in non-Hispanic whites.

Recruitment

The Million Veteran Program (MVP) is a large cohort of fully consented participants who were recruited from the patient populations of 63 Department of Veterans Affairs (VA) medical facilities. Recruitment began in 2011 and is conducted in-person,which is initiated by an invitation letter and completed by answering baseline and lifestyle questionnaires, providing a bloodsample, providing access to medical records, and giving permission for re-contact. Consent to participate is provided after counseling by research staff and mailing of informational materials. All documents and protocols have been approved by the VA Central Institutional Review Board. Blood samples are collected by phlebotomists and banked at the VA Central Biorepository inBoston, MA. Genotyping was conducted using a customized Affymetrix Axiom Biobank Array chip with additional content addedto provide coverage of African and Hispanic haplotypes, as well as markers for common diseases in the VA population. Researchers are provided with de-identified versions of these data, and do not have the ability or authorization to link thesedetails with a participants' identity. While the population is not a representative sample of all individuals living in the US. The sample of individuals is representative ofthe VA population.

Ethics oversight

VA Central Institutional Review Board as well as local IRBs at all VA sites recruiting participants

Note that full information on the approval of the study protocol must also be provided in the manuscript.
